# Visual Object Tracking in First Person Vision

**DOI:** 10.1007/s11263-022-01694-6

**Published:** 2022-10-18

**Authors:** Matteo Dunnhofer, Antonino Furnari, Giovanni Maria Farinella, Christian Micheloni

**Affiliations:** 1 Machine Learning and Perception Lab, University of Udine, Via delle Scienze 206, 33100 Udine, Italy; 2 Image Processing Laboratory, University of Catania, Viale A. Doria 6, 95125 Catania, Italy

**Keywords:** First person vision, Egocentric vision, Visual object tracking, Single object tracking

## Abstract

The understanding of human-object interactions is fundamental in First Person Vision (FPV). Visual tracking algorithms which follow the objects manipulated by the camera wearer can provide useful information to effectively model such interactions. In the last years, the computer vision community has significantly improved the performance of tracking algorithms for a large variety of target objects and scenarios. Despite a few previous attempts to exploit trackers in the FPV domain, a methodical analysis of the performance of state-of-the-art trackers is still missing. This research gap raises the question of whether current solutions can be used “off-the-shelf” or more domain-specific investigations should be carried out. This paper aims to provide answers to such questions. We present the first systematic investigation of single object tracking in FPV. Our study extensively analyses the performance of 42 algorithms including generic object trackers and baseline FPV-specific trackers. The analysis is carried out by focusing on different aspects of the FPV setting, introducing new performance measures, and in relation to FPV-specific tasks. The study is made possible through the introduction of TREK-150, a novel benchmark dataset composed of 150 densely annotated video sequences. Our results show that object tracking in FPV poses new challenges to current visual trackers. We highlight the factors causing such behavior and point out possible research directions. Despite their difficulties, we prove that trackers bring benefits to FPV downstream tasks requiring short-term object tracking. We expect that generic object tracking will gain popularity in FPV as new and FPV-specific methodologies are investigated.

**Supplementary Information:**

The online version contains supplementary material available at 10.1007/s11263-022-01694-6.

## Introduction

First Person Vision (FPV) refers to the study and development of computer vision techniques considering images and videos acquired from a camera mounted on the head of a person—which is referred to as the camera wearer. This setting allows machines to perceive the surrounding environment from a point of view that is the most similar to the one of human beings. In the FPV domain, understanding the interactions between a camera wearer and the surrounding objects is a fundamental problem (Bertasius et al., 2017a, b; Cao et al., 2020; Cai et al., 2016; Damen et al., 2018; Damen et al., 2016; Furnari & Farinella 2020; Grauman 2022; Liu et al., 2020; Ragusa et al., 2020; Wang et al., 2020). To model such interactions, the continuous knowledge of where an object of interest is located inside the video frame is advantageous. Indeed, keeping track of object locations over time allows to understand which objects are moving, which of them are passively captured while not interacted, and how the user relates to the scene.

The benefits of tracking in FPV have been explored by a few previous works in the literature.

**Fig. 1:**
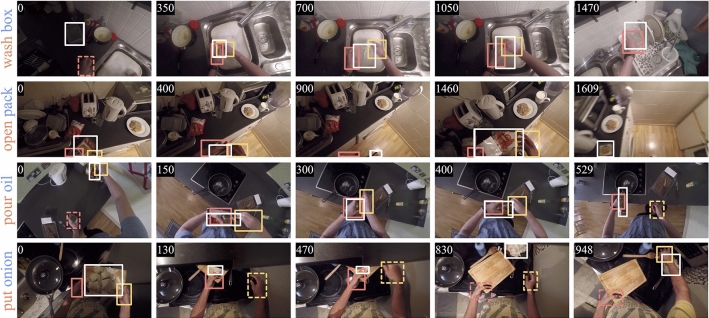
In this paper, we study the problem of visual object tracking in the context of FPV. To achieve such a goal, we introduce a new benchmark dataset named TREK-150, of which some qualitative examples of sequences are represented in this Figure. In each frame, the white rectangle represents the ground-truth bounding box of the target object. The orange and yellow boxes localize left and right hands respectively (plain lines indicate the interaction between the hand and the target). Each number in the top left corner reports the frame index. For each sequence, the action performed by the camera wearer is also reported (verb in orange, noun in blue). As can be noted, objects undergo significant appearance and state changes due to the manipulation by the camera wearer, which makes the proposed setting challenging for current trackers.

For example, visual trackers have been exploited in solutions to comprehend social interactions through faces (Aghaei et al., 2016a, b; Grauman et al., 2022), to improve the performance of hand detection for rehabilitation purposes (Visee et al., 2020), to capture hand movements for action recognition (Kapidis et al., 2019), and to forecast human-object interactions through the analysis of hand trajectories (Liu et al., 2020). Such applications have been made possible trough the development of customized tracking approaches to track specific target categories like people (Alletto et al., 2015; Nigam & Rameshan, 2017), people faces (Aghaei et al., 2016a; Grauman et al., 2022), or hands (Kapidis et al., 2019; Han et al., 2020; Liu et al., 2020; Mueller et al., 2017; Sun et al., 2010; Visee et al., 2020) from a first person perspective.

Despite the aforementioned attempts to leverage tracking in egocentric vision pipelines, the standard approach to *generic-object continuous localisation* in FPV tasks still relies on detection models that evaluate video frames independently (Damen et al., 2018, 2021; Furnari & Farinella, 2020; Ma et al., 2016; Rodin et al., 2021; Sener et al., 2020; Wang et al., 2020; Wu et al., 2019). This paradigm has the drawback of ignoring all the temporal information coming from the object appearance and motion contained in consecutive video frames. Also, it generally requires a higher computational cost due to the need to repeat the detection process in every frame. In contrast, visual object tracking aims to exploit past information about the target to infer its position and shape in the next frames of a video (Maggio & Cavallaro, 2011; Smeulders et al., 2014). This process can improve the efficiency of algorithmic pipelines because of the reduced computational resources needed, but most importantly because it allows to maintain the spatial and temporal reference to specific object instances.

Visually tracking a *generic object* in an automatic way introduces several different challenges that include occlusions, pose or scale changes, appearance variations, and fast motion. The computer vision community has made significant progress in the development of algorithms capable of tracking arbitrary objects in unconstrained scenarios affected by those issues. The advancements have been possible thanks to the development of new and effective tracking principles (Bolme et al., 2010; Bertinetto et al., 2016b; Bhat et al., 2019; Dai et al., 2020; Danelljan et al., 2017a; Henriques et al., 2015; Guo et al., 2021; Zhang et al., 2020; Yan et al., 2021), and to the careful design of benchmark datasets (Fan et al., 2019; Galoogahi et al., 2017; Huang et al., 2019; Li et al., 2016; Mueller et al., 2016; Wu et al., 2015) and competitions (Kristan et al., 2017, 2019, 2020, 2021) that well represent the aforementioned challenging situations. However, all these research endeavours have taken into account mainly the classic third person scenario in which the target objects are passively observed from an external point of view and where they do not interact with the camera wearer. It is a matter of fact that the nature of images and videos acquired from the first person viewpoint is inherently different from the type of image captured from video cameras set as on an external point of view. As we will show in this paper, the particular characteristics of FPV, such as the interaction between the camera wearer and the objects as well as the proximity of the scene and the camera’s point of view, cause the aforementioned challenges to occur with a *different nature and distribution*, resulting in the persistent occlusion, significant scale and state changes of objects, as well as an increased presence of motion blur and fast motion (see Fig. 1).

While the use cases of object tracking in egocentric vision are manifold and the benefit of tracking generic objects is clear as previously discussed, it is evident that visual object tracking is still not a dominant technology in FPV. Only very recent FPV pipelines are starting to employ generic object trackers (Grauman et al., 2022; Rai et al., 2021), but a solution specifically designed to track generic objects in first person videos is still missing. We think this lack of interest towards visual object tracking in FPV is mainly due to the *limited amount of knowledge* present in the literature about the capabilities of current visual object trackers in FPV videos. Indeed, this gap in the research opens many questions about the impact of the first person viewpoint on visual trackers: can the trackers available nowadays be used “off-the-shelf”? How does FPV impact current methodologies? Which tracking approaches work better in FPV scenarios? What factors influence the most the tracking performance? What is the contribution of trackers in FPV? We believe that the particular setting offered by FPV deserves a dedicated analysis that is still missing in the literature, and we argue that further research on this problem cannot be pursued without a thorough study on the impact of FPV on tracking.

In this paper, we aim to extensively analyze the problem of visual object tracking in the FPV domain in order to answer the aforementioned questions. Given the lack of suitable benchmarks, we follow the standard practice of the visual tracking community that suggests to build a curated dataset for evaluation (Galoogahi et al., 2017; Kristan et al., 2019; Liang et al., 2015; Li et al., 2016; Lukezic et al., 2019; Mueller et al., 2016; Wu et al., 2015). Hence, we propose a novel visual tracking benchmark, TREK-150 (TRacking-Epic-Kitchens-150), which is obtained from the large and challenging FPV dataset EPIC-KITCHENS (EK) (Damen et al., 2018, 2021). TREK-150 provides 150 video sequences which we densely annotated with the bounding boxes of a single target object the camera wearer interacts with. The dense localization of the person’s hands and the interaction state between those and the target are also provided. Additionally, each sequence has been labeled with attributes that identify the visual changes the object is undergoing, the class of the target object, as well as the action he/she is performing. By exploiting the dataset, we present an extensive and in-depth study of the accuracy and speed performance of 38 established generic object trackers and of 4 newly introduced baseline FPV trackers. We leverage standard evaluation protocols and metrics and propose new ones. This is done in order to evaluate the capabilities of the trackers in relation to specific FPV scenarios. Furthermore, we assess the trackers’ performance by evaluating their impact on the FPV-specific downstream task of human-object interaction detection.

In sum, the *main contribution* of this manuscript is the first systematic analysis of visual object tracking in FPV. In addition to that, our study brings additional innovations:

(i) The description and release of the new TREK-150 dataset, which offers new challenges and complementary features with respect to existing visual tracking benchmarks;
(ii) A new measure to assess the tracker’s ability to maintain temporal reference to targets;
(iii) A protocol to evaluate the performance of trackers with respect to a downstream task;
(iv) Four FPV baseline trackers, two based on FPV object detectors and two combining such detectors with a state-of-the-art generic object tracker.

Our results show that FPV offers new and challenging tracking scenarios for the most recent and accurate trackers (Dai et al., 2020; Danelljan et al., 2019, 2017a; Song et al., 2018; Wang et al., 2021) and even for FPV trackers. We study the factors causing such performance and highlight possible future research directions. Despite the difficulties introduced by FPV, we prove that trackers bring benefits to FPV downstream tasks requiring short-term object tracking such as hand-object interaction. Given our results and considering the potential impact in FPV, we expect that generic object tracking will gain popularity in this domain as new and FPV-specific methodologies are investigated.^1^

## Related Work

### Visual Tracking in FPV

There have been some attempts to tackle visual tracking in FPV.  Alletto et al. (2015) improved the TLD tracker (Kalal et al., 2012) with a 3D odometry-based module to track people. For a similar task,  Nigam and Rameshan (2017) proposed EgoTracker, a combination of the Struck (Hare et al., 2016) and MEEM (Zhang et al., 2014) trackers with a person re-identification module. Face tracking was tackled by Aghaei et al. (2016a) through a multi-object tracking approach termed extended-bag-of-tracklets. Hand tracking was studied in several works (Han et al., 2020; Kapidis et al., 2019; Mueller et al., 2017; Visee et al., 2020; Sun et al., 2010). Sun et al. (2010) developed a particle filter framework for hand pose tracking. Mueller et al. (2017) instead proposed a solution based on an RGB camera and a depth sensor, while Kapidis et al. (2019) and Visee et al. (2020) combined the YOLO (Redmon et al., 2016) detector trained for hand detection with a visual tracker. The former work used the multi-object tracker DeepSORT (Wojke et al., 2018), whereas the latter employed the KCF (Henriques et al., 2015) single object tracker. Han et al. (2020) exploited a detection-by-tracking approach on video frames acquired with 4 fisheye cameras.

All the aforementioned solutions focused on tracking specific targets (i.e., people, faces, or hands), and thus they are likely to fail in generalizing to arbitrary target objects. Moreover, they have been validated on custom designed datasets, which limits the reproducibility of the works and the ability to compare them to other solutions. In contrast, we focus on the evaluation of algorithms for the generic object tracking task. We design our evaluation to be reproducible and extendable by releasing TREK-150, a set of 150 videos of different objects, which we believe will be useful to study object tracking in FPV. To the best of our knowledge, ours is the first attempt to evaluate systematically and in-depth generic object tracking in FPV.

### Visual Tracking for Generic Settings

In recent years, there has been an increased interest in developing accurate and robust tracking algorithms for generic objects and domains. Preliminary trackers were based on mean shift algorithms (Comaniciu et al., 2000), key-point (Maresca & Petrosino, 2013), part-based methods (Čehovin et al., 2013; Nam et al., 2014), or SVM (Hare et al., 2016) and incremental (Ross et al., 2008) learning. Later, solutions based on correlation filters gained popularity thanks to their processing speed (Bolme et al., 2010; Bertinetto et al., 2016a; Danelljan et al., 2017b; Henriques et al., 2015; Kiani Galoogahi et al., 2017). More recently, algorithms based on deep learning have been proposed to extract efficient image and object features. This kind of representation has been used in deep regression networks (Dunnhofer et al., 2021; Held et al., 2016), online tracking-by-detection methods (Nam & Han, 2016; Song et al., 2018), approaches based on reinforcement learning (Dunnhofer et al., 2019; Yun et al., 2017), deep discriminative correlation filters (Bhat et al., 2019, 2020; Danelljan et al., 2017a, 2019, 2020; Lukežič et al., 2020), trackers based on siamese networks (Bertinetto et al., 2016b; Guo et al., 2021; Li et al., 2019; Wang et al., 2019; Zhang et al., 2020), and more recently in trackers built up on transformer architectures (Chen et al., 2021; Wang et al., 2021; Yan et al., 2021). All these methods have been designed for tracking arbitrary target objects in unconstrained domains. However, no solution has been studied and validated on a number of diverse FPV sequences as we propose in this paper.

### FPV Datasets and Tasks

Different datasets are currently available in the FPV community for the study of particular tasks. The CMU dataset (De la Torre et al., 2009) was introduced for studying the recognition of the actions performed by the camera wearer. Videos belonging to this dataset are annotated with labels expressing only the actions performed (up to 31) by the person, and they comprise around 200K frames. The EGTEA Gaze+ dataset (Li et al., 2018) extended the FPV scenarios represented in the previous dataset by providing 2.4 M frames. Similarly as (De la Torre et al., 2009), only labels for the actions performed by the camera wearer have been associated to the videos. In addition to the action labels, the ADL dataset (Pirsiavash & Ramanan, 2012) introduced around 137K annotations in the form of bounding boxes for the localization of the objects involved in the actions. Other than for the action recognition task, the MECCANO dataset (Ragusa et al., 2020) was aimed to study active object detection and recognition as well as hand-object interaction. The dataset is designed to represent an industrial-like scenario and provides 299K frames, 64K bounding-boxes, 60 action labels, and 20 object categories. The EPIC-KITCHENS dataset (Damen et al., 2018, 2021) is currently one of the largest and most representative datasets available for vision-based tasks based on an egocentric point of view. It is composed of 20 M frames and provides annotations for action recognition, action anticipation, and object detection.

Despite the extensive amount of labels for different FPV tasks, all the aforementioned datasets (Damen et al., 2018, 2021; Pirsiavash & Ramanan, 2012; Ragusa et al., 2020) do not offer annotations to study object tracking. This is because the available bounding boxes for the localization of objects are not relative to the specific instances of the objects but only to their categories. Such kind of annotations does not allow to distinguish different objects of the same category when these appear together in the images. Furthermore, such datasets provide only sparse annotations (typically at 1/2 FPS) and they do not provide tracking-specific annotations (Müller et al., 2018; Kristan et al., 2017; Wu et al., 2015). Hence, they cannot be used for an accurate and in-depth evaluation of trackers in FPV. To the best of our knowledge, our proposed TREK-150 dataset is the first tool that provides the chance of studying in-depth the visual object tracking task in the context of first-person viewpoint egocentric videos. In addition, with the release of dense annotations for the position of the camera wearer’s hands, for the state of interaction between hands and the target object, and for the action performed by the camera wearer, TREK-150 is suitable to analyze the visual tracking task in relation to all those FPV-specific tasks that require continuous and dense object localization (e.g. human-object interaction).

### Visual Tracking Benchmarks

Disparate bounding-box level benchmarks are available today to evaluate the performance of single-object visual tracking algorithms. The Object Tracking Benchmarks (OTB) OTB-50 (Wu et al., 2013) and OTB-100 (Wu et al., 2015) are two of the most popular benchmarks in the visual tracking community. They provide 51 and 100 sequences respectively, including generic target objects like vehicles, people, faces, toys, characters, etc. The Temple-Color 128 (TC-128) dataset (Liang et al., 2015) comprises 128 videos that were acquired for the evaluation of color-enhanced trackers. The UAV123 dataset (Mueller et al., 2016) was constructed to benchmark the tracking progress on videos captured by unmanned aerial vehicles (UAVs) cameras. The 123 videos included in this benchmark represent 9 different classes of target. The NUS-PRO dataset (Li et al., 2016) contains 365 sequences and aims to benchmark human and rigid object tracking with targets belonging to one of 8 categories. The Need for Speed (NfS) dataset (Galoogahi et al., 2017) provides 100 sequences with a frame rate of 240 FPS. The aim of the authors was to benchmark the effects of frame rate variations on the tracking performance. The VOT2019 benchmark (Kristan et al., 2019) was the last iteration of the annual Visual Object Tracking challenge that required bounding-boxes as target object representation. This dataset contains 60 highly challenging videos, with generic target objects belonging to 30 different categories. The Color and Depth Tracking Benchmark (CDTB) dataset (Lukezic et al., 2019) offers 80 RGB sequences paired with a depth channel. This benchmark aims to explore the use of depth information to improve tracking. The Transparent Object Tracking Benchmark (TOTB) (Fan et al., 2021) provides 225 videos of transparent target objects, and has been introduced to study the robustness of trackers to the particular appearance of such kind of objects.

Following the increased development of deep learning-based trackers, large-scale generic-domain tracking datasets have been recently released (Müller et al., 2018; Huang et al., 2019; Fan et al., 2021). These include more than a thousand videos normally split into training and test subsets. The evaluation protocol associated with these sets requires the evaluation of the trackers after they have been trained on the provided training set.

Even though all the presented benchmarks offer various tracking scenarios, and some of them may include videos acquired from a first person point of view, no one was specifically designed for tracking in FPV. Moreover, since in this paper we aim to benchmark the performance of visual object trackers regardless of their approach, we follow the practice of previous works (Fan et al., 2021; Galoogahi et al., 2017; Kristan et al., 2019; Li et al., 2016; Liang et al., 2015; Lukezic et al., 2019; Mueller et al., 2016; Wu et al., 2015) and set up a well representative and described dataset for evaluation. We believe that TREK-150 is useful for the tracking community because it offers different tracking situations and new target object categories that are not present in other tracking benchmarks.

## The TREK-150 Benchmark

In this section, we describe TREK-150, the novel dataset proposed for the study of the visual object tracking task in FPV. TREK-150 is composed of 150 video sequences. In each sequence, a single target object is labeled with a bounding box which encloses the appearance of the object in each frame in which the object is visible (as a whole or in part). Every sequence is additionally labeled with attributes describing the visual variability of the target and the scene in the sequence. To study the performance of trackers in the setting of human-object interaction, we provide bounding box localization of hands and labels for their state of interaction with the target object. Moreover, two additional verb and noun attributes are provided to indicate the action performed by the person and the class of the target, respectively. Some qualitative examples of the video sequences with the relative annotations are shown in Fig. 1. Table 1 reports key statistics of our dataset in comparison with existing tracker evaluation benchmarks. It is worth noticing that the proposed dataset is competitive in terms of size with respect to the evaluation benchmarks available in the visual (single) object tracking community.

We remark that TREK-150 has been designed for the *evaluation* of visual tracking algorithms in FPV regardless of their methodology. Indeed, in this paper, we do not aim to provide a large-scale dataset for the development of deep learning-based trackers. Instead, our goal is to assess the impact of the first-person viewpoint on current trackers. To achieve this goal we follow the standard practice in the visual object tracking community (Fan et al., 2021; Galoogahi et al., 2017; Kristan et al., 2019; Liang et al., 2015; Li et al., 2016; Lukezic et al., 2019; Mueller et al., 2016; Wu et al., 2015) that suggests to set up a small but *well described dataset* to benchmark the tracking progress.

**Table 1:** Statistics of the proposed TREK-150 benchmark compared with other benchmarks designed for single visual object tracking evaluation

Benchmark	OTB-50	OTB-100	TC-128	UAV123	NUS-PRO	NfS	VOT2019	CDTB	TOTB	GOT-10k*	LaSOT*	TREK-150
	(Wu et al., 2013)	(Wu et al., 2015)	(Liang et al., 2015)	(Mueller et al., 2016)	(Li et al., 2016)	(Galoogahi et al., 2017)	(Kristan et al., 2019)	(Lukezic et al., 2019)	(Fan et al., 2021)	(Huang et al., 2019)	(Fan et al., 2019)	
# Videos	51	100	128	123	365	100	60	80	225	180	280	150
# Frames	29K	59K	55K	113K	135K	383K	20K	102K	86K	23K	685k	97K
Min frames across videos	71	71	71	109	146	169	41	406	126	51	1000	161
Mean frames across videos	578	590	429	915	371	3830	332	1274	381	127	2448	649
Median frames across videos	392	393	365	882	300	2448	258	1179	389	100	2102	484
Max frames across videos	3872	3872	3872	3085	5040	20,665	1500	2501	500	920	9999	4640
Frame rate	30 FPS	30 FPS	30 FPS	30 FPS	30 FPS	240 FPS	30 FPS	30 FPS	30 FPS	10 FPS	30 FPS	60 FPS
# Target object classes	10	16	27	9	8	17	30	23	15	84	70	34
# Sequence attributes	11	11	11	12	12	9	6	13	12	6	14	17
Target absent labels	$\times$	$\times$	$\times$	$\times$	$\times$	$\times$	$\checkmark$	$\checkmark$	$\checkmark$	$\checkmark$	$\checkmark$	$\checkmark$
Labels for the interaction with the target	$\times$	$\times$	$\times$	$\times$	$\times$	$\times$	$\times$	$\times$	$\times$	$\times$	$\times$	$\checkmark$
FPV	$\times$	$\times$	$\times$	$\times$	$\times$	$\times$	$\times$	$\times$	$\times$	$\times$	$\times$	$\checkmark$
# Action verbs	n/a	n/a	n/a	n/a	n/a	n/a	n/a	n/a	n/a	n/a	n/a	20

For the datasets marked with * we report the statistics of their test set

### Data Collection

#### Video Collection

The videos contained in TREK-150 have been sampled from EK (Damen et al., 2018, 2021), which is a public, large-scale, and diverse dataset of egocentric videos focused on human-object interactions in kitchens. This is currently one of the largest datasets for understanding human-object interactions in FPV. Thanks to its dimension, EK provides a significant amount of diverse interaction situations between various people and several different types of objects. Hence, it allows us to select suitable disparate tracking sequences that reflect the common scenarios tackled in FPV tasks. EK offers videos annotated with the actions performed by the camera wearer in the form of temporal bounds and verb-noun labels. The subset of EK known as EK-55 (Damen et al., 2018) also contains sparse bounding box references of manipulated objects annotated at 2 frames per second in a temporal window around each action. We exploited such a feature to obtain a suitable pool of video sequences interesting for object tracking. Particularly, we cross-referenced the original verb-noun temporal annotations of EK-55 to the sparse bounding box labels. This allowed to select sequences in which the camera wearer manipulates an object during an action. Each sequence is composed of the video frames contained within the temporal bounds of the action, extracted at the original 60 FPS frame rate and at the original full HD frame size (Damen et al., 2018, 2021). From the initial pool, we selected 150 video sequences which were characterized by attributes such as scale changes, partial/full occlusion and fast motion, which are commonly considered in standard tracking benchmarks (Fan et al., 2019; Kristan et al., 2019; Mueller et al., 2016; Müller et al., 2018; Wu et al., 2015). The top part of Table 2 reports the 13 attributes considered for the selection.

### Data Labeling

#### Single Object Tracking

In this study, we restricted our analysis to the tracking of a single target object per video. This has been done because in the FPV scenario a person interacts through his/her hands with one object at a time in general (Damen et al., 2018, 2021). If a person interacts with two objects at the same time those can be still tracked by two single object trackers. Moreover, focusing on a single object allows us to analyze better all the challenging and relevant factors that characterize the tracking problem in FPV. We believe that future work could investigate the employment of multiple object tracking (MOT) (Dendorfer et al., 2021; Luiten et al., 2021) solutions for a general understanding of the position and movement of all objects visible in the scene. We think the in-depth study presented in this paper will give useful insights for the development of such methods.

**Table 2:** Selected sequence attributes

Attribute	Meaning
SC	*Scale change* the ratio of the bounding-box area of the first and the current frame is outside the range [0.5, 2]
ARC	*Aspect ratio change* the ratio of the bounding-box aspect ratio of the first and the current frame is outside the range [0.5, 2]
IV	*Illumination variation* the area of the target bounding-box is subject to light variation
SOB	*Similar objects* there are objects in the video of the same object category or with similar appearance to the target
RIG	*Rigid object* the target is a rigid object
DEF	*Deformable object* the target is a deformable object
ROT	*Rotation* the target rotates in the video
POC	*Partial occlusion* the target is partially occluded in the video
FOC	*Full occlusion* the target is fully occluded in the video
OUT	*Out of view* the target completely leaves the video frame
MB	*Motion blur* the target region is blurred due to target or camera motion
FM	*Fast motion* the target bounding-box has a motion change larger than its size
LR	*Low resolution* the area of the target bounding-box is less than 1000 pixels in at least one frame
HR	*High resolution* the area of the target bounding-box is larger than 250,000 pixels in at least one frame
HM	*Head motion* the person moves their head significantly thus causing camera motion
1 H	*1 Hand interaction* the person interacts with the target object with one hand for consecutive video frames
2 H	*2 Hands interaction* the person interacts with the target object with both hands for consecutive video frames

The first block of rows describes attributes commonly used by the visual tracking community. The last four rows describe additional attributes introduced in this paper to characterize FPV tracking sequences

**Fig. 2:**
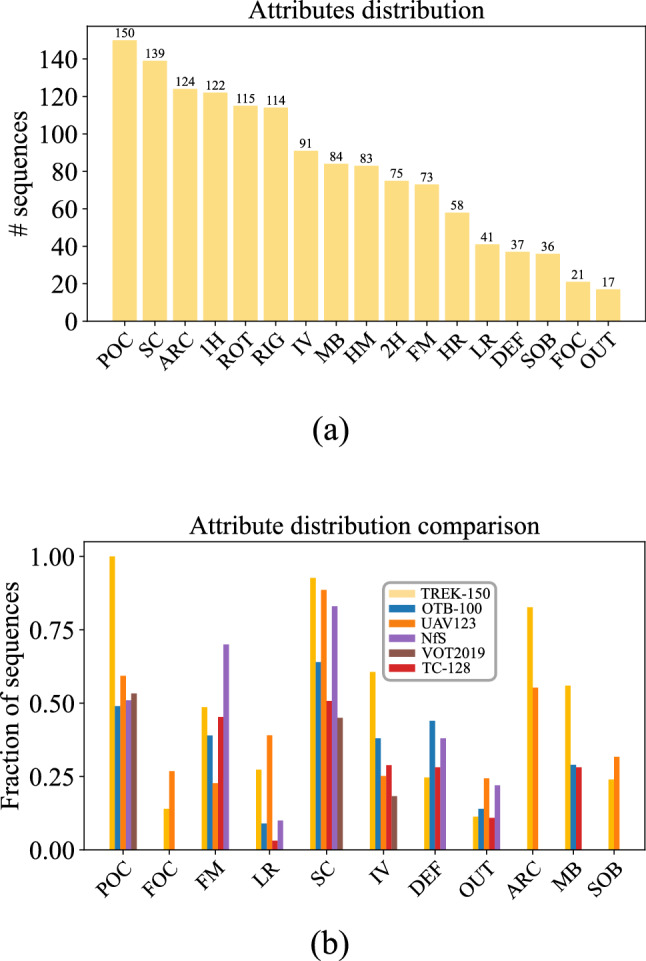
**a** Distribution of the sequences within TREK-150 with respect to the attributes used to categorize the visual variability happening on the target object and scene. **b** Comparison of the distributions of common sequence attributes across different benchmarks

**Fig. 3:**
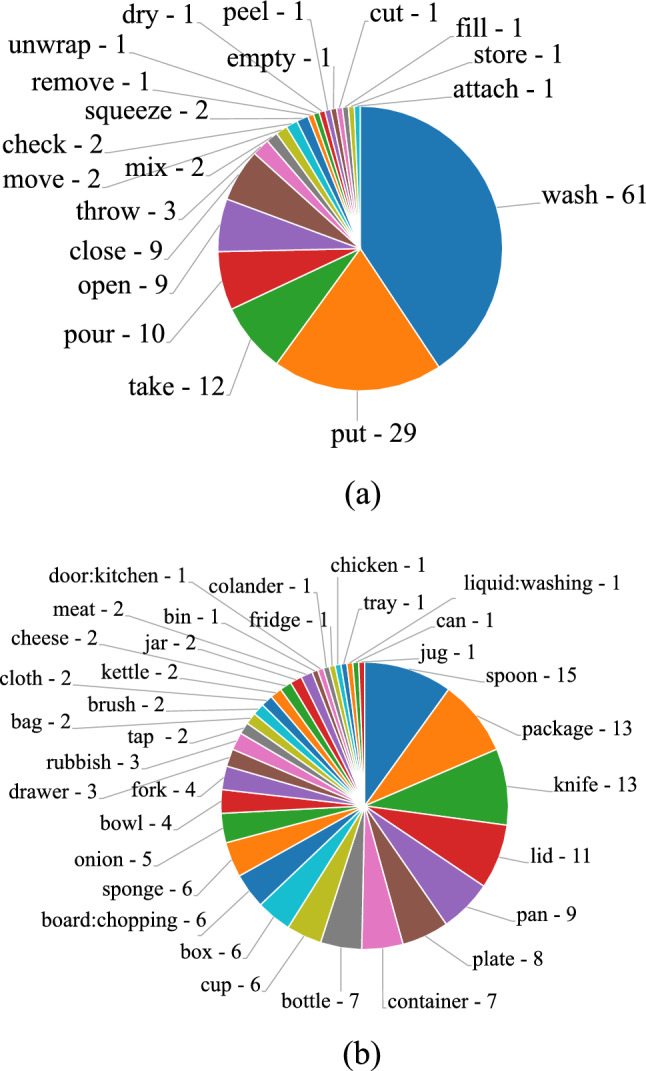
Distributions of **a** action verb labels and **b** target object categories

#### Frame-Level Annotations

After selection, the 150 sequences were associated to only 3000 bounding boxes, due to the sparse nature of the object annotations in EK-55. Since it has been shown that visual tracking benchmarks require dense and accurate box annotations (Fan et al., 2019; Kristan et al., 2019; Mueller et al., 2016; Valmadre et al., 2018), we re-annotated the bounding boxes of the target objects on the 150 sequences selected. Batches of sequences were delivered to annotators (21 subjects) who were instructed to perform the labeling. Such initial annotations were then carefully checked and refined by a PhD student, and finally revised by an early-stage researcher and by two professors. This process produced 97,296 frames labeled with bounding boxes related to the position and visual presence of objects the camera wearer is interacting with. Following the initial annotations of EK-55, we employed axis-aligned bounding boxes to localize the target objects. This design choice is supported by the fact that such a representation is largely used in many FPV pipelines (Furnari & Farinella, 2020; Furnari et al., 2017; Furnari & Farinella, 2019; Damen et al., 2018; Kapidis et al., 2019; Shan et al., 2020; Visee et al., 2020). Therefore, computing tracking metrics based on such representations allows us to correlate the results with those of object localization pipelines in FPV tasks, ultimately better highlighting the impact of trackers in such contexts. Also, the usage of more sophisticated target representation would have restricted our analysis since the majority of state-of-the-art trackers output just axis-aligned bounding boxes (Bertinetto et al. 2016a, b; Bhat et al., 2019; 2020; Bolme et al., 2010; Chen et al., 2020; Dai et al., 2020; Danelljan et al. Danelljan et al. 2017a, b, 2019, 2020; Fu et al. 2021; Guo et al., 2021; Held et al., 2016; Henriques et al., 2015; Huang et al., 2020; Kiani Galoogahi et al., 2017; Li et al., 2018, 2019; Nam & Han, 2016; Park & Berg, 2018; Song et al., 2018; Wang et al., 2018, 2021; Xu et al., 2020; Yan et al., 2019, 2021; Zhang & Peng, 2019; Zhang et al., 2020), and their recent progress on various benchmarks using such representation (Wu et al., 2015; Mueller et al., 2016; Galoogahi et al., 2017; Lukezic et al., 2019; Fan et al., 2021; Müller et al., 2018; Fan et al., 2019; Huang et al., 2019) proves that it provides sufficient information for tracker initialization and consistent and reliable performance evaluation. Moreover, we point out that many of the objects commonly appearing in FPV scenarios are difficult to annotate consistently with more sophisticated target representations.footref We remark that the proposed bounding boxes have been carefully and tightly drawn around the visible parts of the objects. Figure 13 of the supplementary document shows some examples of the quality of the bounding-box annotations of TREK-150 in contrast to the ones available in the popular OTB-100 tracking benchmark.

In addition to the bounding boxes for the object to be tracked, TREK-150 provides per-frame annotations of the location of the left and right hand of the camera wearer and of the state of interaction happening between each hand and the target object. Interaction annotations consist of labels expressing which hand of the camera wearer is currently in contact with the target object (e.g., we used the labels LHI, RHI, BHI to express whether the person is interacting with the target by her/his left or right hand or with both hands). We considered an interaction happening even in the presence of an object acting as a medium between the hand and the target. E.g., we considered the camera wearer to interact with a dish even if a sponge is in between her/his hand and the dish. The fourth row of Fig. 1 shows a visual example of these situations. These kinds of annotations have been obtained by the manual refinement (performed by the four aforementioned subjects) of the output given by the FPV hand-object interaction detector Hands-in-Contact (HiC) (Shan et al., 2020). In total, 166,883 hand bounding boxes (82,678 for the left hand, 84,205 for the right hand) and 77,993 interaction state labels (24,466 for interaction with left hand, 16,171 with right hand, 37,356 with both hands) are present in TREK-150.

#### Sequence-Level Annotations

The sequences have been also labeled considering 17 attributes which define the motion and visual appearance changes the target object or the scene is subject to. These are used to analyze the performance of the trackers under different aspects that may influence their execution. The attributes employed in this study include 13 attributes used in standard tracking benchmarks (Fan et al., 2019; Müller et al., 2018; Wu et al., 2015), plus 4 additional new ones (High Resolution, Head Motion, 1-Hand Interaction, 2-Hands Interaction) which have been introduced in this paper to characterize sequences from FPV-specific point of views. The 17 attributes are defined in Table 2. Fig. 2a reports the distributions of the sequences with respect to the 17 attributes, while Fig. 2b compares the distributions of the most common attributes in the field in TREK-150 and in other well-known tracking benchmarks. Our dataset provides a larger number of sequences affected by partial occlusions (POC), changes in scale (SC) and/or aspect ratio (ARC), motion blur (MB), and illumination variation (IV). These peculiarities are due to the particular first person viewpoint and to the human-object interactions which affect the camera motion and the appearance of objects. Based on the verb-noun labels of EK, sequences were also associated to 20 verb labels (e.g., “wash”—see Fig. 1) and 34 noun labels indicating the category of the target object (e.g., “box”). Fig. 3a–b report the distributions of the videos with respect to verb and target object labels. As can be noted, our benchmark reflects the long-tail distribution of labels in EK (Damen et al., 2018).

**Table 3:** Characteristics of the generic object trackers considered in our evaluation

Tracker	Venue	Image representation				Matching operation						Model update	Class given by Lukezic et al. (2020)				Offline training dataset
		Pixel	HOG	Color	CNN	CF	CC	Concat	T-by-D	Had	Tra		$\text {ST}_0$	$\text {ST}_1$	$\text {LT}_0$	$\text {LT}_1$	
MOSSE (Bolme et al., 2010)	CVPR 2010	$\checkmark$				$\checkmark$						$\checkmark$	$\checkmark$				
DSST (Danelljan et al., 2017b)	BMVC 2014	$\checkmark$	$\checkmark$			$\checkmark$						$\checkmark$	$\checkmark$				
KCF (Henriques et al., 2015)	TPAMI 2015		$\checkmark$			$\checkmark$						$\checkmark$	$\checkmark$				
MDNet (Nam & Han, 2016)	CVPR 2016				VGG-M				$\checkmark$			$\checkmark$		$\checkmark$			I, O, IV
Staple (Bertinetto et al., 2016a)	CVPR 2016		$\checkmark$	$\checkmark$		$\checkmark$						$\checkmark$	$\checkmark$				
SiamFC (Bertinetto et al., 2016b)	ECCVW 2016				AlexNet		$\checkmark$						$\checkmark$				G
GOTURN (Held et al., 2016)	ECCV 2016				AlexNet			$\checkmark$					$\checkmark$				ID, A
ECO (Danelljan et al., 2017a)	CVPR 2017				VGG-M	$\checkmark$						$\checkmark$		$\checkmark$			
BACF (Kiani Galoogahi et al., 2017)	ICCV 2017		$\checkmark$			$\checkmark$						$\checkmark$	$\checkmark$				
DCFNet (Wang et al., 2017)	ArXiv 2017				VGG-M	$\checkmark$						$\checkmark$	$\checkmark$				
VITAL (Song et al., 2018)	CVPR 2018				VGG-M				$\checkmark$			$\checkmark$		$\checkmark$			I, O, IV
STRCF (Li et al., 2018)	CVPR 2018		$\checkmark$			$\checkmark$						$\checkmark$	$\checkmark$				
MCCTH (Wang et al., 2018)	CVPR 2018		$\checkmark$	$\checkmark$		$\checkmark$						$\checkmark$	$\checkmark$				
DSLT (Lu et al., 2018)	ECCV 2018				VGG-16		$\checkmark$					$\checkmark$	$\checkmark$				ID, IV, C
MetaCrest (Park & Berg, 2018)	ECCV 2018				VGG-M	$\checkmark$						$\checkmark$		$\checkmark$			I, ID, V
SiamRPN++ (Li et al., 2019)	CVPR 2019				ResNet-50		$\checkmark$						$\checkmark$				I, C, ID, IV, Y
SiamMask (Wang et al., 2019)	CVPR 2019				ResNet-50		$\checkmark$						$\checkmark$				I, ID, YV
SiamDW (Zhang & Peng, 2019)	CVPR 2019				ResNet-22		$\checkmark$						$\checkmark$				I, ID, Y
ATOM (Danelljan et al., 2019)	CVPR 2019				ResNet-18	$\checkmark$						$\checkmark$		$\checkmark$			I, C, L, T
DiMP (Bhat et al., 2019)	ICCV 2019				ResNet-50	$\checkmark$						$\checkmark$		$\checkmark$			I, C, L, T, G
SPLT (Yan et al., 2019)	ICCV 2019				ResNet-50	$\checkmark$						$\checkmark$				$\checkmark$	IV, ID
UpdateNet (Zhang et al., 2019)	ICCV 2019				AlexNet		$\checkmark$					$\checkmark$	$\checkmark$				L
SiamFC++ (Xu et al., 2020)	AAAI 2020				AlexNet		$\checkmark$						$\checkmark$				Y, ID, IV, C, G
GlobalTrack (Huang et al., 2020)	AAAI 2020				ResNet-50					$\checkmark$					$\checkmark$		C, G, L
PrDiMP (Danelljan et al., 2020)	CVPR 2020				ResNet-50	$\checkmark$						$\checkmark$		$\checkmark$			I, C, L, T, G
SiamBAN (Chen et al., 2020)	CVPR 2020				ResNet-50		$\checkmark$						$\checkmark$				IV, ID, C, G, L, Y
D3S (Lukežič et al., 2020)	CVPR 2020				ResNet-50	$\checkmark$							$\checkmark$				YV
LTMU (Dai et al., 2020)	CVPR 2020				ResNet-50	$\checkmark$	$\checkmark$		$\checkmark$			$\checkmark$				$\checkmark$	I, L
Ocean (Zhang et al., 2020)	ECCV 2020				ResNet-50		$\checkmark$						$\checkmark$				Y, ID, IV, C, G
KYS (Bhat et al., 2020)	ECCV 2020				ResNet-50	$\checkmark$						$\checkmark$		$\checkmark$			T, L, G
TRASFUST (Dunnhofer et al., 2020)	ACCV 2020				ResNet-18			$\checkmark$					$\checkmark$				G
SiamGAT (Guo et al., 2021)	CVPR 2021				ResNet-50		$\checkmark$						$\checkmark$				Y, ID, IV, C, G
TrDiMP (Wang et al., 2021)	CVPR 2021				ResNet-50	$\checkmark$					$\checkmark$	$\checkmark$		$\checkmark$			C, L, T, G
LightTrack (Yan et al., 2021)	CVPR 2021				NAS		$\checkmark$						$\checkmark$				Y, ID, IV, C, G
TransT (Chen et al., 2021)	CVPR 2021				ResNet-50						$\checkmark$	$\checkmark$					C, L, T, G
STMTrack (Fu et al., 2021)	CVPR 2021				GoogLeNet			$\checkmark$				$\checkmark$	$\checkmark$				G
STARK (Yan et al., 2021)	ICCV 2021				ResNet-50						$\checkmark$	$\checkmark$		$\checkmark$	$\checkmark$		C, L, T, G
KeepTrack (Mayer et al., 2021)	ICCV 2021				ResNet-50	$\checkmark$						$\checkmark$		$\checkmark$	$\checkmark$		I, C, L, T, G

We provide details about: the Image Representation employed by the trackers (Pixel column—$\checkmark$ if the tracker uses raw pixel intensity values; HOG column—$\checkmark$ if the tracker uses Histogram of Oriented Gradients; Color column—$\checkmark$ if the tracker uses Color Names or Intensity; CNN column—the Convolutional Neural Network backbone used); the Matching Operation performed to find the target in sequence frames (CF column—$\checkmark$ if the tracker uses correlation filters; CC column—$\checkmark$ if the tracker uses the cross correlation; Concat column—$\checkmark$ if the tracker concatenates features; T-by-D column—$\checkmark$ if the tracker uses a tracking-by-detection approach; Had column—$\checkmark$ if the tracker uses hadamard correlation; Tra column—$\checkmark$ if the tracker uses a transformer-based correlation). The $\checkmark$ symbol in the Model Update column expresses whether the tracker updates the target model during the tracking procedure. The next four columns report the category of tracking approach according to Lukezic et al. (2020) ($\text {ST}_0$ column—short-term trackers without any re-detection mechanism; $\text {ST}_1$ column—short-term trackers without any re-detection mechanism but that estimate tracking confidence; $\text {LT}_0$ column—pseudo long-term trackers that do not detect failure and do not perform explicit re-detection; $\text {LT}_1$ column—long-term trackers that detect tracking failure and perform re-detection). The last column presents the datasets used to optimize the tracker in the offline training phase (I—ImageNet (Deng et al., 2009), IV—ILSVRC-VID (Russakovsky et al., 2015), ID—ILSVRC-DET (Russakovsky et al., 2015), C—COCO (Lin et al., 2014), Y—YouTube-BB (Real et al., 2017), YV—YouTube-VOS (Xu et al., 2018), A—ALOV (Smeulders et al., 2014), O—OTB (Wu et al., 2015), V—VOT (Kristan et al., 2016), T—TrackingNet (Müller et al., 2018), G—GOT-10k (Huang et al., 2019), L—LaSOT (Fan et al., 2019))

## Trackers

### Generic Object Trackers

Among the examined trackers, 38 have been selected to represent different popular approaches to generic-object visual tracking. Specifically, in the analysis we have included short-term trackers (Lukezic et al., 2020) based on both correlation-filters with hand-crafted features (MOSSE (Bolme et al., 2010), DSST (Danelljan et al., 2017b), KCF (Henriques et al., 2015), Staple (Bertinetto et al., 2016a), BACF (Kiani Galoogahi et al., 2017), DCFNet (Wang et al., 2017), STRCF (Li et al., 2018), MCCTH (Wang et al., 2018)) and deep features (ECO (Danelljan et al., 2017a), ATOM (Danelljan et al., 2019), DiMP (Bhat et al., 2019), PrDiMP (Danelljan et al., 2020), KYS (Bhat et al., 2020), KeepTrack (Mayer et al., 2021)). We also considered deep siamese networks (SiamFC (Bertinetto et al., 2016b), GOTURN (Held et al., 2016), DSLT (Lu et al., 2018), SiamRPN++ (Li et al., 2019), SiamDW (Zhang & Peng, 2019), UpdateNet (Zhang et al., 2019), SiamFC++ (Xu et al., 2020), SiamBAN (Chen et al., 2020), Ocean (Zhang et al., 2020), SiamGAT (Guo et al., 2021), STMTrack (Fu et al., 2021)), tracking-by-detection methods (MDNet (Nam & Han, 2016), VITAL (Song et al., 2018)), as well as trackers based on target segmentation representations (SiamMask (Wang et al., 2019), D3S (Lukežič et al., 2020)), meta-learning (MetaCrest (Park & Berg, 2018)), fusion of trackers (TRASFUST (Dunnhofer et al., 2020)), neural architecture search (LightTrack (Yan et al., 2021)), and transformers (TrDiMP (Wang et al., 2021), TransT (Chen et al., 2021), STARK (Yan et al., 2021)). The long-term (Lukezic et al., 2020) trackers SPLT (Yan et al., 2019), GlobalTrack (Huang et al., 2020), and LTMU (Dai et al., 2020) have been also taken into account in the study. These kinds of trackers are designed to address longer target occlusion and out of view periods by exploiting an object re-detection module. All of the selected trackers are state-of-the-art approaches published between the years 2010 and 2021. Table 3 reports detailed information about the 38 considered generic-object trackers regarding the: venue and year of publication; type of image representation used; type of target matching strategy; employment of target model updates; and category of tracker according to the classification of (Lukezic et al., 2020). For each tracker, we used the code publicly available and adopted default parameters in order to have a fair comparison between the different tracking methodologies (i.e., to avoid comparisons between trackers specifically optimized for TREK-150 and non-optimized trackers). The original hyper-parameter values lead to the best and most likely generalizable instances of all the trackers. The code was run on a machine with an Intel Xeon E5-2690 v4 @ 2.60GHz CPU, 320 GB of RAM, and an NVIDIA TITAN V GPU.

**Fig. 4:**
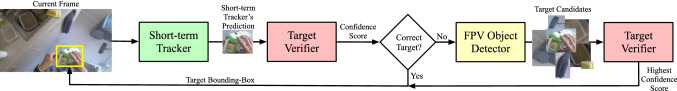
Scheme of execution of the proposed FPV baseline trackers LTMU-F and LTMU-H based on LTMU (Dai et al., 2020)

### FPV Trackers

Since there are no public implementations of the FPV trackers described in Sect. 2.1, we introduce 4 new FPV-specific tracking baselines.

#### TbyD-F/H

The first two FPV baselines build up on FPV-specific object detectors (Damen et al., 2018; Shan et al., 2020). Considering that they are popular approach for object localization in FPV and off-the-shelf FPV-trained instances are publicly available, we tested whether they can be used as naïve tracking baselines. To this end, we define a simple processing procedure which we found to work surprisingly well. At the first frame of a tracking sequence, the initial bounding box is memorized as current information about the target’s object’s position. Then, at every other frame, an FPV object detector is run to provide the boxes of all object instances present in the frame. As output for the current frame, the bounding-box having larger intersection-over-union (IoU) with the previously memorized box is given. If the detector does not output detections for a particular frame or none of its predicted boxes has IoU greater than 0, then the previously memorized box is given as output for the current frame. As object detectors, we used the EK-55 trained Faster-R-CNN (Damen et al., 2018; Ren et al., 2015) and the Faster-R-CNN-based hand-object interaction detector HiC (Shan et al., 2020). The tracking baseline built upon the first detector is referred to as TbyD-F, while the one built on the second as TbyD-H.

#### LTMU-F/H

We developed 2 other FPV-specific trackers in addition to the aforementioned ones. In this case, we wanted to combine the capabilites of generic object trackers with the FPV-specific object localization abilities of detectors (Damen et al., 2018; Shan et al., 2020). Particularly, the baselines combine the LTMU tracker (Dai et al., 2020) with FPV-specific object detectors. The first solution, referred to as LTMU-F, employs the Faster-R-CNN object detector trained on EK-55 (Damen et al., 2018), while the second, denoted as LTMU-H, uses the hand-object detector HiC (Shan et al., 2020). These two trackers exploit the respective detectors as re-detection modules according to the LTMU scheme (Dai et al., 2020). For a better understanding, we briefly recap the processing procedure of the LTMU tracker (Dai et al., 2020). After being initialized with the target in the first frame of a sequence, at every other frame LTMU first executes a short-term tracker that tracks the target in a local area of the frame based on the target’s last position. The patch extracted from the box prediction of the tracker is evaluated by an online-learned verification module based on MDNet (Nam & Han, 2016), which outputs a probability estimate of the target being contained in the patch. Such an estimate in companion with the tracker’s predicted traget presence are used to decide if the short-term tracker is tracking the target or not. If it is, its predicted box is given as output for the current frame. In the other case, a re-detection module is executed to look for the target in the whole frame. The re-detector returns some candidate locations which may contain the target and each of these is checked by the verification module. The candidate patch with the highest confidence is given as output and used as a new target location to re-initialize the short-term tracker. The verifier’s output as well as the tracker’s confidence are used to decide when to update the parameters of the first. Based on experiments, we used STARK (Yan et al., 2021) as short-term tracker and the aforementioned FPV-based detectors as re-detection modules. For LTMU-F, such a module has been set to retain the first 10 among the many detections given as output, considering a ranking based on the scores attributed by the detector to each detection. If no detection is given for a frame, the last available position of the target is considered as a candidate location. For LTMU-H, we used the object localizations of the hand-object interaction detections given by the FPV version of HiC (Shan et al., 2020) as target candidate locations. HiC is implemented as an improved Faster R-CNN which is set to provide, at the same time, the localization of hands and interacted objects, as well as their state of interaction. As for LTMU-F, if no detection is given for a frame, the last available position of the target is considered as a candidate location. For both detection methods, the original pre-trained models provided by the authors have been used. The described setups, the common scheme of which is presented in Fig. 4, give birth to two new FPV trackers that implement conceptually different strategies for FPV-based object localization and tracking. Indeed, the first solution aims to just look for objects in the scene, while the second one reasons in terms of the interaction happening between the camera wearer and the objects.

The choice of using LTMU (Dai et al., 2020) as a baseline methodology stems from its highly modular scheme which makes it the most easily configurable tracker with state-of-the-art performance available today. We took advantage of the commodity of a such framework to insert the FPV-specific modules described before.

## Evaluation Settings

### Evaluation Protocols

The protocols used to execute the trackers are described in the following.

#### One-Pass Evaluation

We employed the one-pass evaluation (OPE) protocol detailed in (Wu et al., 2015) which implements the most realistic way to run a tracker in practice. The protocol consists of two main stages: (i) initializing a tracker with the ground-truth bounding box of the target in the first frame; (ii) letting the tracker run on every subsequent frame until the end of the sequence and record predictions to be considered for the evaluation. To obtain performance scores for each sequence, predictions and ground-truth bounding boxes are compared according to some distance measure only in frames where ground-truths are present (ground-truth bounding boxes are not given for frames in which the target is fully occluded or out of the field of view). The overall scores are obtained by averaging the scores achieved for every sequence.

The tracker initialization with the ground-truth is performed to evaluate the trackers in the best possible conditions, i.e. when accurate information about the target is given. In practical applications, such a user-defined information is generally unavailable. We expect this scenario to occur especially in FPV applications where object localization is obtained via detectors (Damen et al., 2018; Shan et al., 2020). Detectors predict bounding boxes with spatial noise (in the position and/or in the scale), and the initialization of trackers with such a noisy information could influence the tracking performance. Hence, to understand the impact of the initial box given by an object detector, we consider a version of the OPE protocol, referred to as OPE-D, where each tracker is initialized in the first frame in which the detector’s prediction has IoU $\ge$ 0.5 with the ground-truth box. From such a frame (that could be delayed in time with respect to the beginning of the sequence), each tracker is also run with the ground-truth box. The change in the metric values obtained after running the two modalities are used to quantify the impact of the initialization box.

**Fig. 5:**
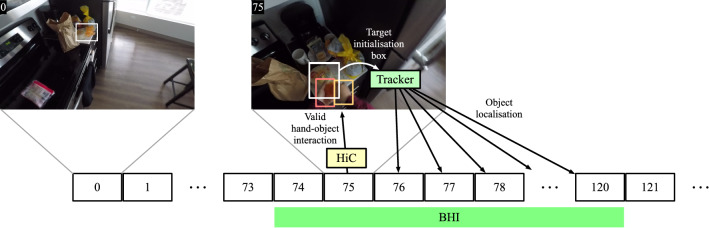
Schematic visualization of the protocol designed to execute trackers in the context of a hand-object interaction (HOI) detection task. The HOI labels provided for TREK-150 are used to consider sub-sequences of frames in which the camera wearer is interacting with the target object. In this picture, the labels BHI are employed to indicate that an interaction by both hands is happening in the frame range [74, 120]. On such sub-sequences, a systematic pipeline for HOI detection and tracking is run. The HOI detector HiC (Shan et al., 2020) is first executed in every frame to obtain a valid HOI (in this example the first valid detection is obtained at frame 75). Once such an event is determined, the tracker is initialized with the bounding box given by HiC for the object involved in the interaction. The tracker is then run on all the subsequent frames to provide the reference to such an object

#### Multi-Start Evaluation

To obtain a more robust evaluation (Kristan et al., 2016), especially for the analysis over sequence attributes and action verbs, we employed the recent protocol proposed in (Kristan et al., 2020), which defines different points of initialization along a video. In more detail, for each sequence, different initialization points—called anchors—separated by 2 s are defined. Anchors are always set in the first and last frames of a sequence. Some of the inner anchors are shifted forward by a few frames in order to avoid frames in which the target is not visible. A tracker is run on each of the sub-sequences yielded by the anchor either forward or backward in time depending on the longest sub-sequence the anchor generates. The tracker is initialized with the ground-truth annotation in the first frame of the sub-sequence and let run until its end. Then, as for the OPE, predicted and ground-truth boxes are compared to obtain performance scores for each sub-sequence. Scores for a single sequence are computed by averaging the scores of each sub-sequence weighted by their length in number of frames. Similarly, the overall scores for the whole dataset are obtained by averaging each sequence’s score weighted by its number of frames. We refer to this protocol as multi-start evaluation (MSE). It allows a tracker to better cover all the situations happening in the sequences, ultimately leading to more robust evaluation scores.

#### Hand-Object Interaction Evaluation

We also evaluated trackers in relation to a video-based hand-object interaction (HOI) detection solution. This is done in order to assess their direct impact on a downstream FPV-specific task. The aim of this problem is to determine when and where in the frames the camera wearer is interacting (e.g., by touching/manipulating) with an object with his/her hands. Considering the requirement of generic object localization (Shan et al., 2020), we think a video-based configuration of such a problem to be a suitable task to exploit visual object trackers. To achieve the goal, we built a solution composed of a HiC instance (Shan et al., 2020) to detect the hands and their state of interaction with an object and a visual tracker to maintain the reference to it. The HiC detector is run at every frame until it finds a valid HOI detection. Such an event is said to occur when the bounding box predictions for the hands have an IoU $\ge 0.5$ with the hand ground-truth boxes, the predicted interaction state is “in contact”, and the object bounding box has an IoU $\ge 0.5$ with the ground-truth box (Shan et al., 2020). Then, the predicted object-related box is used to initialize the tracker, and for the subsequent frames, it is run to provide the localization of that object (that is the one involved in the interaction). A graphical representation of the execution of the described pipeline is given in Fig. 5. Taking inspiration from the metric used by Shan et al. (2020) to evaluate HiC on static images, we quantify the performance of the proposed pipeline by the normalized count of frames in which the given HOI detection matches the ground-truth annotation available. Such matching is said to happen when the bounding box predictions for the hands have an IoU $\ge 0.5$ with the hand ground-truth boxes, the predicted interaction state is “in contact”, and the object bounding box has an IoU $\ge 0.5$ with the ground-truth box (Shan et al., 2020). For our experiments, we restricted the analysis of the solution on the sub-sequences contained in TREK-150 in which an HOI is present. These are determined by considering the sub-sequences of consecutive frames having the same interaction label (i.e., LHI, RHI, BHI). To obtain an overall performance score, which we refer to as Recall, we average the sub-sequence scores after having them weighted by the sub-sequence lengths in number of frames, in a similar fashion as we did to compute score in the MSE. To evaluate the impact of visual trackers on this task, we switch the pipeline’s tracker with each of the ones studied in this work. This experimental procedure gives us an estimate of the accuracy of the HOI detection system under configurations with different trackers. More interestingly, the proposed evaluation protocol allows also to build a ranking of the trackers based on the results of a downstream application. To the best of our knowledge, this setup brings a new way to assess the performance of visual object trackers.

#### Real-Time Evaluation

Since many FPV tasks such as object interaction (Damen et al., 2016) and early action recognition (Furnari & Farinella, 2019), or action anticipation (Damen et al., 2018), require real-time computation, we evaluate trackers in such a setting by following the instructions given in (Kristan et al., 2017; Li et al., 2020). Explanations and results are given in the supplementary document.

### Performance Measures

**Fig. 6:**
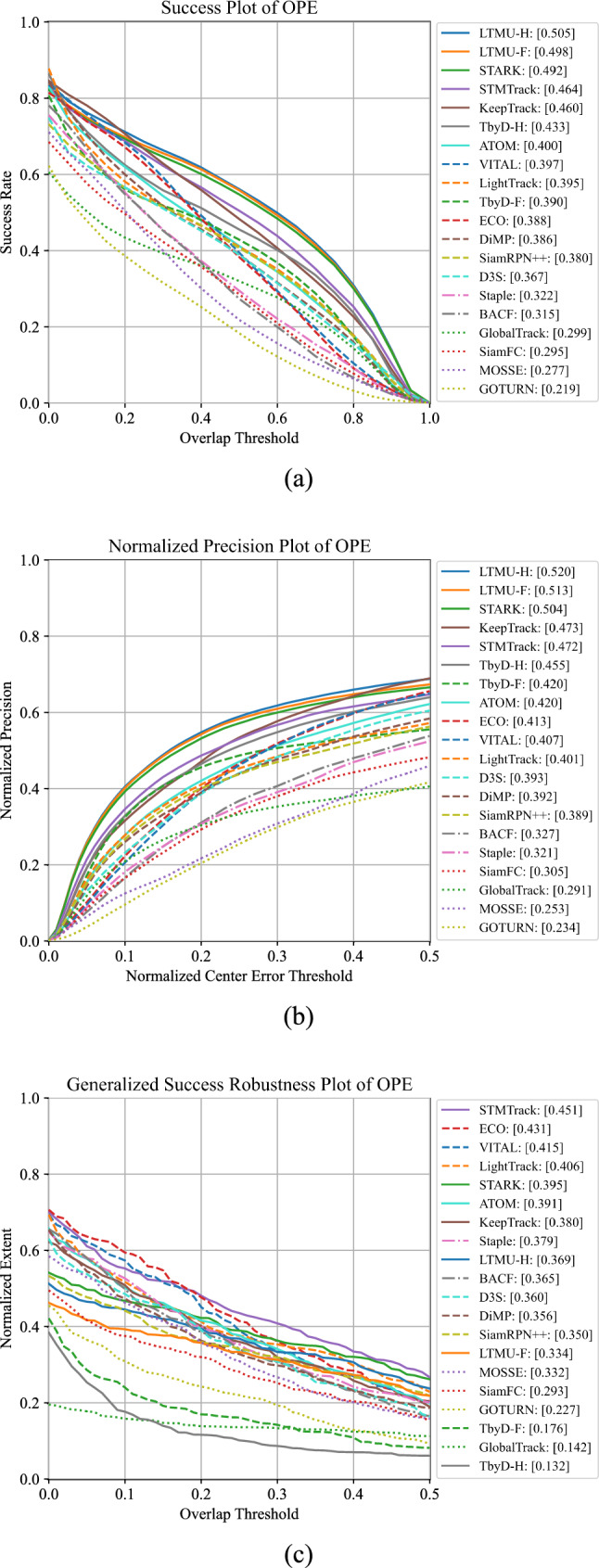
Performance of 20 of the 42 selected trackers on the proposed TREK-150 benchmark under the OPE protocol. In brackets, next to the trackers’ names, we report the SS, NPS, and GSR values

To quantify the performance of the trackers, we used different measures that compare trackers’ predicted bounding boxes with the temporally aligned ground-truth boxes. To evaluate the overall localization accuracy of the trackers, we employ the success plot (Wu et al., 2015), which shows the percentage of predicted boxes whose IoU with the ground-truth is larger than a threshold varied from 0 to 1 (Fig. 6a). We also use the normalized precision plot (Müller et al., 2018), that reports, for a variety of thresholds, the percentage of boxes whose center points are within a given normalized distance from the ground-truth (Fig. 6b). As summary measures, we report the success score (SS) (Wu et al., 2015) and normalized precision scores (NPS) (Müller et al., 2018), which are computed as the Area Under the Curve (AUC) of the success plot and normalized precision plot respectively.

Along with these standard metrics, we employ a novel plot which we refer to as generalized success robustness plot (Fig. 6c). For this, we take inspiration from the robustness metric proposed in Kristan et al. (2020) which measures the normalized extent of a tracking sequence before a failure. We believe this aspect to be especially important in FPV as a superior ability of a tracker to maintain longer references to targets can lead to the better modeling of actions and interactions. The original metric proposed in Kristan et al. (2020) uses a fixed threshold of 0.1 on the bounding box overlap to detect a collapse of the tracker. Such a value was determined mainly to reduce the chance of cheating in the VOT2020 competition and it is not necessarily the case that such a value could work well for different tracking applications. To generalize the metric, we take inspiration from the success and normalized precision plots and propose to use different box overlap thresholds ranging in [0, 0.5] to determine the collapse. We consider 0.5 as the maximum threshold as higher overlaps are usually associated to positive predictions in many computer vision tasks. Overall, our proposed plot allows to assess the length of tracking sequences in a more general way that is better aligned with the requirements of different application scenarios including FPV ones. Similarly to Wu et al. (2015); Müller et al. (2018), we use the AUC of the generalized success robustness plot to obtain an aggregate score which we refer to as generalized success robustness (GSR).

**Fig. 7:**
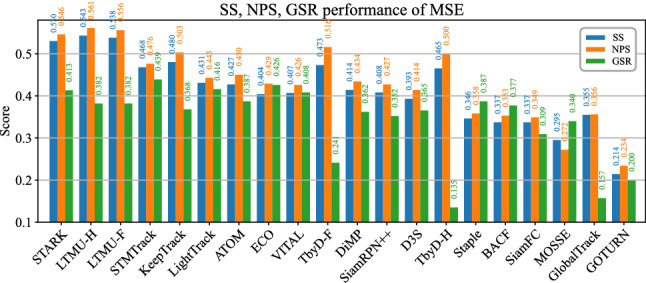
SS, NPS, and GSR performance of 20 of the 42 benchmarked generic object trackers on the proposed TREK-150 benchmark achieved under the MSE protocol. The trackers are ordered by the average value of their SS, NPS, GSR scores

**Fig. 8:**
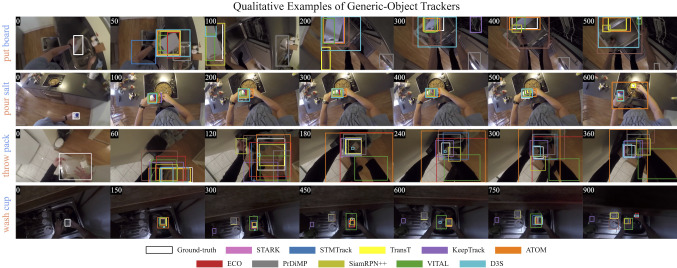
Qualitative results of some of the generic object trackers benchmarked on the proposed TREK-150 dataset

## Results

In this section, we discuss the outcomes of our proposed study. For a better readability, in Figures and Tables we provide results for 20 of the 42 studied trackers. The results for all the trackers are given in the Figures and Tables of the supplementary document.

### Performance of Generic Object Trackers

Figures 6 and 16 report the performance of the generic object trackers on TREK-150 using the OPE protocol, while Figs. 7 and 17 present the results achieved with the MSE protocol. Figure 8 presents examples that qualitatively show the performance of some of the trackers. Considering the results on a tracking approach basis, we have that trackers based on deep learning (e.g. STARK, TransT, KeepTrack, LTMU, TrDiMP, ATOM, VITAL, ECO, Ocean) perform better than those based on hand-crafted features (e.g. BACF, MCCTH, DSST, KCF). Among the first class of trackers, the ones leveraging online adaptation mechanisms (e.g. STARK, STMTrack, KeepTrack, LTMU, TrDiMP, ATOM, VITAL, ECO, KYS, DiMP) are more accurate than the ones based on single-shot instances (e.g. SiamGAT, Ocean, D3S, SiamBAN, SiamRPN++) Trackers based on the transformer architecture (Vaswani et al., 2017) (e.g. STARK, TransT, TrDiMP) hold the highest positions in the rankings of all the plots, suggesting that the representation learning and matching approach exploited by such trackers is suitable for better target-background discrimination in the FPV setting. Indeed, the transformer-based matching operation between template and searching areas like the one implemented by STARK and TransT leads to a higher bounding box overlap on average (SS performance of Fig. 6a) and to a better centered bounding box (NPS performance of Fig. 6b).

Generally, the generalized success robustness plot in Fig. 6c and the GSR results of Fig. 7 report different rankings of the trackers, showing that more spatially accurate trackers are not always able to maintain their accuracy for longer periods of time. Trackers that aim to build robust target models via online methods (e.g. STMTrack, ECO, TrDiMP, VITAL, MDNet, ATOM) result in better solutions for keeping longer temporal reference to objects. Particularly, the results achieved by STMTrack tell that a strategy based on memory networks building a highly dynamic representation of the template during tracking is beneficial to maintain a longer reference to the target.

By comparing the performance of the selected trackers with the results they achieve on standard benchmarks such as OTB-100 (Wu et al., 2015), as reported in Fig. 18 of the supplementary document, it can be noticed that the overall performance of all the trackers is decreased across all measures when considering the FPV scenario. Considering the extended usage of data driven approaches (e.g. deep learning) in visual tracking nowadays, we assessed the impact of leveraging large-scale FPV object localization data for training. In-depth discussion and results are provided in Section 11.3 of the supplementary document. In short, some methodologies such as deep discriminative trackers (Bhat et al., 2019) benefit from FPV-specific data, but the overall tracking performance still does not reach the quality that is observed in more common tracking benchmarks (Wu et al., 2015; Mueller et al., 2016; Galoogahi et al., 2017; Kristan et al., 2019). Other methodologies such as siamese network-based trackers (Li et al., 2019) and transformer-based trackers (Yan et al., 2021) are not able to exploit the context of FPV from still FPV images. This weakness could be improved by yet-to-come large-scale FPV tracking datasets. Overall, these outcomes demonstrate that, for the current availability of tracking data as well as the visual tracking knowledge in exploiting such, the FPV setting poses new challenges to present trackers. It is worth mentioning that our achieved conclusions are consistent with the demonstrated performance drop of other object localization models (e.g. object detection) exploited between classical domains (Everingham et al., 2015; Lin et al., 2014) and FPV domains (Damen et al., 2018).

**Table 4:** OPE and MSE performance of the baseline FPV-based tracking-by-detection methods TbyD-H and TbyD-F under different configurations

Tracker	Version	OPE			MSE		
		SS	NPS	GSR	SS	NPS	GSR
TbyD-F	SORT	0.313	0.310	0.311	0.347	0.350	0.338
	IoU w prev box	0.390	0.420	0.176	0.473	0.516	0.241
	IoU w prev box + SORT	0.390	0.425	0.192	0.476	0.521	0.264
TbyD-H	SORT	0.237	0.213	0.222	0.264	0.252	0.241
	IoU w prev box	0.433	0.455	0.132	0.465	0.500	0.135
	IoU w prev box + SORT	0.432	0.457	0.137	0.465	0.502	0.142

**Table 5:** Performance of the proposed baseline FPV-trackers LTMU-H and LTMU-F applied over different trackers and under the OPE and MSE protocols used for the evaluation on TREK-150

Tracker	Version	OPE			MSE		
		SS	NPS	GSR	SS	NPS	GSR
DiMP-MU	Baseline	0.411	0.432	0.320	0.445	0.469	0.342
	LTMU-F	0.456	0.477	0.372	0.485	0.508	0.375
	LTMU-H	0.461	0.486	0.376	0.495	0.517	0.380
STMTrack	Baseline	0.464	0.472	0.451	0.468	0.476	0.439
	LTMU-F	0.461	0.471	0.408	0.471	0.481	0.411
	LTMU-H	0.487	0.499	0.438	0.498	0.509	0.429
STARK	Baseline	0.492	0.504	0.395	0.530	0.546	0.413
	LTMU-F	0.498	0.513	0.334	0.538	0.556	0.382
	LTMU-H	0.505	0.520	0.370	0.543	0.561	0.382

### Performance of the FPV-Specific Trackers

The results achieved by the proposed TbyD-F and TbyD-H FPV-based tracking-by-detection baselines are compared with the generic object trackers in Figs. 6, 16 and 7, 17. As can be noticed, the baselines have competitive results with the best trackers in the SS and NPS metrics, but they struggle in the GSR. This means that they are not able to maintain reference to the objects even though the other scores suggest they provide spatially accurate localizations. By comparing TbyD-F with TbyD-H, we observe that the second is better in an OPE-like execution scenario, while the first achieves higher scores in the MSE experiments. Table 4 reports the performance of such two trackers with other strategies (details are given in Section 9.1 of the supplementary document) that implement target association on top of object detection (Bewley et al., 2016; Dave et al., 2020). A simple application of SORT (Bewley et al., 2016) does not work as well as demonstrated in other domains (Dave et al., 2020), and applying such method in combination with the strategy described in Sect. 4.2.1 brings little benefit.

Figures 6, 16 and 7, 17 also show the performances of the other FPV baselines LTMU-F and LTMU-H in comparison with the different trackers. In both the OPE and MSE experiments, the proposed trackers achieve the top spots in the SS and NPS rankings, while they lose some performance in the GSR score. Table 5 shows the performance gain in applying the LTMU-F/H scheme over different generic object trackers (Dai et al., 2020; Fu et al., 2021; Yan et al., 2021). Overall, both LTMU-F and LTMU-H increase the SS and NPS metrics of the underlying tracker, with the second presenting a generally larger improvement. In the versions with STARK and STMTrack, the GSR scores are decreased. However, looking at the DiMP-MU version (as used in Dai et al. (2020)) we see that the performance is improved by a good margin in all the metrics, including the GSR. Considering that such an underlying tracker uses a MetaUpdater (Dai et al., 2020) to better assess the consistency of the tracker in triggering re-detection and model update, we hypothesize that such a module could bring benefit to the other versions if properly customized to. Fig. 20 of the supplementary document presents some qualitative examples of the performance of the LTMU-F/H trackers in contrast to the baseline one. Overall, the message to take from these outcomes is that adapting a state-of-the-art method with FPV-specific components allows to increase the tracking performance. Combining hand and object tracking, as the baseline LTMU-H naïvely does, results a promising direction. We hence expect significant performance improvements to be achievable by a tracker accurately designed to exploit FPV-specific cues such as the characteristics of the interaction between the target and the camera wearer.

### Initialization by an Object Detector

**Fig. 9:**
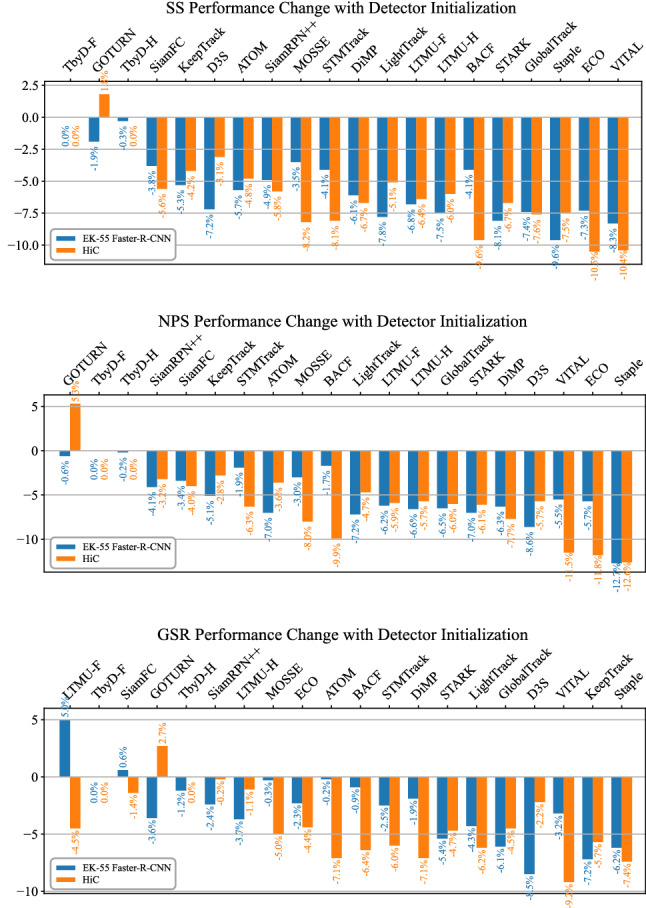
Results of the OPE-D experiment in which the bounding box for initialization is given either by the EK-55 trained Faster-R-CNN (Damen et al., 2018) or the HiC detector (Shan et al., 2020). The performance change (in percentage) for 20 of the selected trackers with respect with the ground-truth initialization is reported for the SS, NPS, and GSR metrics. The trackers are ordered by the average performance change

Figures 9, 19 report the SS, NPS, GSR performance change when the EK-55 Faster-R-CNN (Damen et al., 2018) or the HiC (Shan et al., 2020) detection bounding box is used to initialize the trackers. In general, such a process causes a drop in the tracking performance. This can be explained by the noise in the position and scale of the initial target state that consequently affects the constructions of the models that are used for tracking during the video (Wu et al., 2015). By computing the average delta across the trackers for each of the metrics, we obtain that Faster-R-CNN causes SS, NPS, GSR drops of $-$5.3%, $-$5.1%, $-$3.1%. HiC leads to slightly larger drops of $-$5.9%, $-$5.7%, $-$4.4%. It is worth mentioning that Faster-R-CNN provided 149 valid detections out of 150 with an average delay of 14 frames from the start of the sequence, while HiC gave 146 valid detections with a delay of 28 frames. Hence, HiC is a weaker object detector. Overall, we consider the average performance drop quite limited, thus making the trackers usable even in cases of noisy initialization. TbyD-F/H are among the trackers losing less accuracy, but despite this their performance does not surpass trackers more susceptible to noise, such as LTMU-F/H, STARK, TransT. Indeed, when initialized by Faster-R-CNN, TbyD-H achieves SS 0.440, while LTMU-H, STARK, and TransT, achieve SS 0.478, 0.470, 0.466, respectively.

**Fig. 10:**
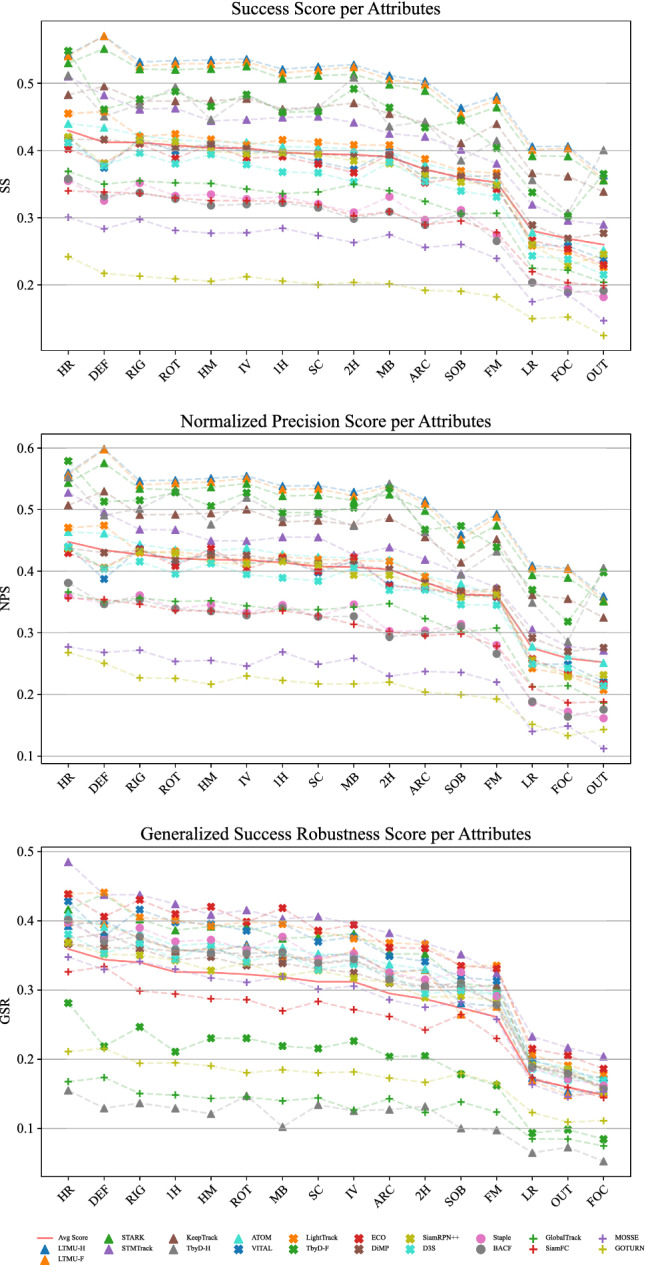
SS, NPS, and GSR performance achieved under the MSE protocol of 20 the 42 selected trackers with respect to the sequence attributes available in TREK-150. (The results for the POC attribute are not reported because this attribute is present in every sequence). The red plain line highlights the average tracker performance

**Fig. 11:**
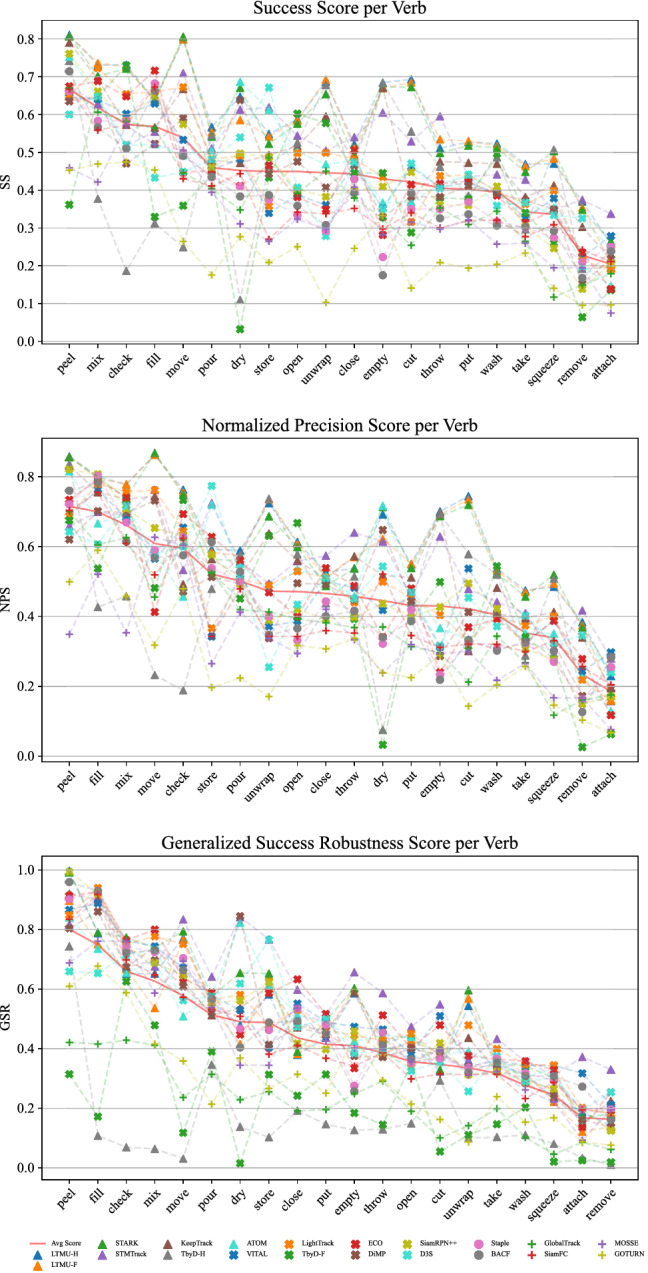
SS, NPS, and GSR performance achieved under the MSE protocol of 20 of the 42 selected trackers with respect to the action verbs performed by the camera wearer and available in TREK-150. The red plain line highlights the average performance

**Fig. 12:**
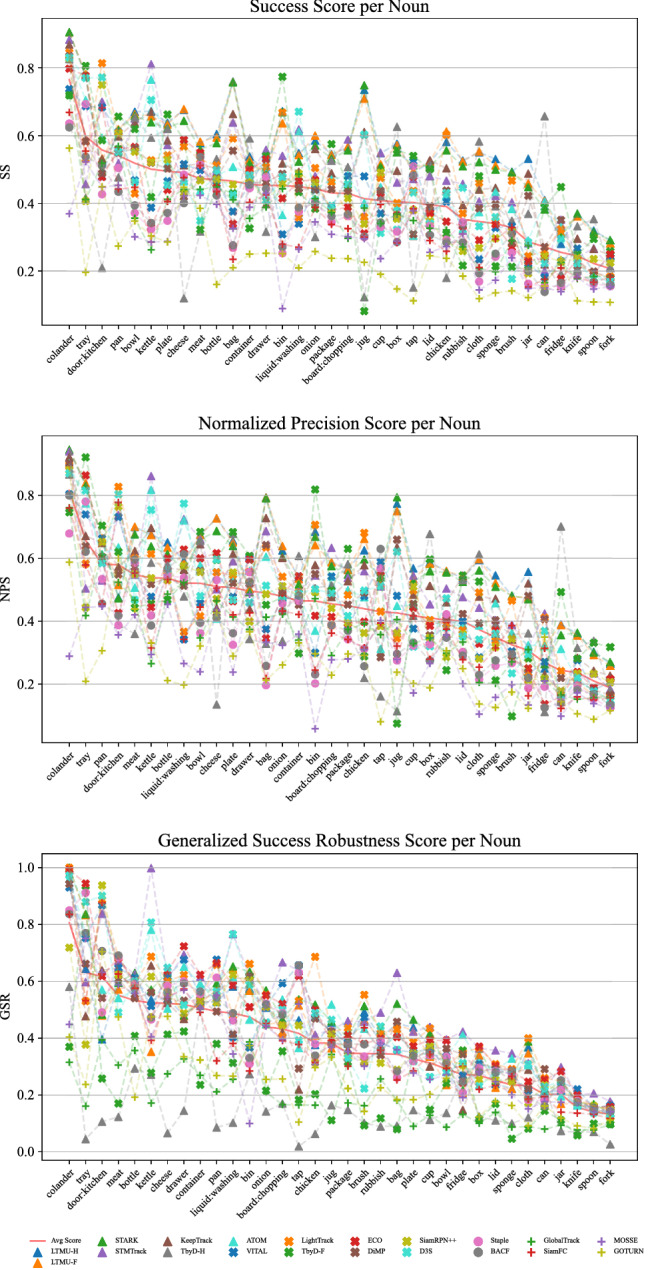
SS, NPS, and GSR performance achieved under the MSE protocol of 20 the 42 selected trackers with respect to the target noun categories available in TREK-150. The red plain line highlights the average tracker performance

### Attribute Analysis

Figure 10 reports the SS, NPS, and GSR scores, computed with the MSE protocol, of the 20 representative trackers with respect to the attributes introduced in Table 2. We do not report results for the POC attribute as it is present in every sequence, as shown in Fig. 2a. It stands out clearly that full occlusion (FOC), out of view (OUT) and the small size of targets (LR) are the most difficult situations for all the trackers. The fast motion of targets (FM) and the presence of similar objects (SOB) are also critical factors that cause drops in performance. Rotations (ROT) and the illumination variation (IV) are better addressed by the trackers. The algorithms also do not demonstrate significant behavior changes between the tracking of rigid or deformable objects. With respect to the new 4 sequence attributes related to FPV, the results report that tracking objects held with two hands (2 H) is more difficult than tracking objects held with a single hand (1 H). This is because the manipulation of the target by two hands generates situations in which the occlusions are more extended over the object’s appearance. Trackers are instead quite robust to the head motion (HM), which influences the camera movements, and seem to cope well with objects appearing in larger sizes (HR).

In terms of algorithmic principles, we have that STARK has better SS results over the second-best generic object tracker, TransT, across all the conditions described by the attributes except for the case of deformable objects (DEF) and the presence of similar objects (SOB). In the latter situations, the performance of the two trackers is around the same. For the NPS, STARK results better than TransT in general, even though the gap between them is reduced. TransT outputs better centered bounding boxes in the DEF and SOB conditions. Considering the GSR measure, we observe that STMTrack results in the best methodology across most of the attributes. The improvement over the other solutions is particularly significant in the presence of the challenging conditions of small objects (LR), target out-of-view (OUT), and full occlusion (FOC). STMTrack exhibits also a much better score with objects appearing in large size (HR). The ECO tracker instead provides longer references to targets in the case of head motion (HM), motion blur (MB), and fast motion (FM). With respect to the introduced FPV trackers, we have that the performance of STARK is improved by LTMU-H and LTMU-F overall. The TbyD-H tracker has a particularly higher SS and NPS performance in out of view conditions (OUT), suggesting a capability in finding again the targets after the re-apperance in the scene. These outcomes tell that the introduced FPV-specific components are particularly helpful in the circumstances that affect the trackers the most.

### Action Analysis

The plot in Fig. 11 reports the MSE protocol results of SS, NPS, and GSR with respect to the action verb labels associated to the actions performed by the camera wearer in each video sequence. We think that the results presented in the following can give cues about the exploitation of trackers for action recognition tasks. In general, we observe that the actions mainly causing a spatial displacement of the target (e.g. “move”, “store”, “check”) have less impact on the performance of the trackers. Instead, actions that change the state, shape, or aspect ratio of the target object (e.g. “remove”, “squeeze”, “cut”, “attach”) generate harder tracking scenarios. Also the sequences characterized by the “wash” action verb lead trackers to poor performance. Indeed, such an action makes the object harder to track because of the many occlusions caused by the persistent and severe manipulation washing involves. It can be noted from the plots that no tracker prevails overall, but LTMU-F/H, STARK, and TransT occupy the top stops especially in the plots relative to SS and NPS. In general, the performance of the trackers varies much across the different actions showing that various approaches are suitable to track under the different conditions generated.

The plots in Fig. 12 presents the performance scores of the trackers with respect to the target noun labels, i.e. the categories of target object. Rigid, regular-sized objects such as “pan”, “kettle”, “bowl”, “plate”, and “bottle” are among the ones associated with higher average SS greater or around 0.5, but some of them (e.g. “plate” and “bottle”) lead to lower GSR scores meaning that trackers provide a spatially accurate but short temporal reference to such kind of objects. In contrast, other rigid objects such as “knife”, “spoon”, “fork” and “can” are more difficult to track from the point of view of all the considered measures (the scores are around 0.3 or lower). This is probably due to the particularly thin shape of these objects and the light reflectance they are easily subject to. Deformable objects such as “sponge”, “onion”, “cloth” and “rubbish” are in general also difficult to track.

### Hand-Object Interaction Evaluation

Tables 6 and 7 present the results of the evaluation of the HOI task described in Sect. 5.1.3 in relation to the considered trackers. Despite we are showing that FPV introduces challenges for current trackers, with this experiment we want to assess whether they can be still exploited in the FPV domain to obtain information about the objects’ locations and movements in the scene (Furnari et al., 2017; Furnari & Farinella, 2020; Sener et al., 2020; Shan et al., 2020; Wang et al., 2020). The results given in the first column of the table report the Recall of the proposed video-based HOI detection pipeline in which each tracker is included. The values in the brackets of the second column report the SS, NPS, and GSR results achieved by the tracker run in an OPE-like fashion on the same sub-sequences on which the pipeline is executed. It can be noticed how the performance difference between the trackers is reduced with respect to what showed in Figs. 6 and 16. This demonstrates that when deployed for HOI, the different tracking methodologies lead to an overall similar pipeline. Particularly, it results that STARK is a better suited methodology for tracking objects starting from an initialization given by an object detection algorithm in this context. By comparing the Recall with the tracker performance scores (SS, NPS, GSR), it can be noted that there is a correlation between the first and the SS, since the ranking of the trackers according to the first measure is very similar to the one of the second measure.

In Table 8 of the supplementary document, the results of an oracle-based solution that gives the optimal bounding box for the interacted object at the first frame of HOI are presented. The first thing that stands out is the performance gap with respect to what reported in Tables 6 and 7. This is due to the performance of HiC which struggles to find a valid HOI detection in the proposed video-based pipeline. This issue delays the initialization of the tracker making the overall pipeline not detecting and localizing the HOI in many frames. These outcomes show that, if initialized with a proper bounding box for the object involved in the interaction, the trackers are able to maintain the spatial and temporal reference to such an object for all the interaction period with promising accuracy. Indeed, the Recall values achieved by the proposed HOI system with LTMU-H reaches 0.754. It is also worth observing that the SS, NPS, GSR scores achieved in this experiment reflect the performance achieved by the trackers with the OPE protocol on the full sequences of TREK-150, as reported in Figs. 6 and 16. These results demonstrate that the evaluation of the trackers’ performance on the original sequences of TREK-150 can lead to conclusions about the behavior of the trackers in particular FPV application scenarios. Furthermore, the reader might wonder why there is such a large absolute difference in the values of the SS, NPS, and GSR present in Table 8 and those in the brackets of Fig. 16. This can be explained by the fact that in the considered HOI evaluation the lengths of the video sequences are very short (the average length is of 81 frames). In contrast, the average length of the full video sequences present in TREK-150 is 649 frames, which is much higher than the previously discussed number. Such a shorter duration of the videos simplifies the job of the trackers since the variations of the target object and the scene are less significant in these conditions rather than in longer sequences. A justification to this explanation is also given by the GSR results of Figs. 6 and 16. For example, on such measure, STARK achieves 0.395 which means that such an algorithm tracks successfully until the 39.5% of a sequence length. In number of frames, such a fraction is 256 on average. This value is much higher than the length of the sub-sequences and explains why the performance of STARK is so successful in the context of this FPV application. Furthermore, in the oracle-based HOI experiment we observe that the ranking of the trackers slightly changes. Trackers that reached lower spots in this experimental setting (e.g. TbyD-H, LTMU, Ocean, D3S), in the HiC-based pipeline compete in making the HOI system more accurate (i.e. they increase the Recall). Considering that in the latter situation the initialization box is not as accurate as the ground-truth, such an outcome additionally confirms that the different trackers are subject in a different manner to the initialization noise.

**Table 6:** Results of the experiment in which 20 of the considered trackers are evaluated by the Recall of an FPV HOI detection pipeline where trackers are used as localization method for the object involved in the interaction

Tracker	Recall	(SS, NPS, GSR)
STARK	**0.248**	(**0.211**, *0.221*, ***0.222***)
LTMU-H	***0.246***	(***0.210***, ***0.222***, 0.217)
LTMU-F	*0.245*	(***0.210***, *0.221*, 0.216)
TbyD-H	0.238	(*0.205*, **0.223**, 0.163)
LightTrack	0.233	(0.197, 0.212, **0.228**)
KeepTrack	0.232	(0.201, 0.214, 0.212)
SiamRPN++	0.227	(0.191, 0.206, 0.209)
TbyD-F	0.220	(0.184, 0.202, 0.179)
STMTrack	0.216	(0.196, 0.202, *0.219*)
D3S	0.211	(0.187, 0.199, 0.208)
ECO	0.211	(0.181, 0.196, 0.217)
DiMP	0.210	(0.186, 0.198, 0.211)
ATOM	0.207	(0.186, 0.198, 0.213)
VITAL	0.198	(0.178, 0.192, 0.213)
SiamFC	0.195	(0.171, 0.180, 0.195)
GlobalTrack	0.195	(0.170, 0.180, 0.144)
BACF	0.188	(0.170, 0.189, 0.206)
Staple	0.182	(0.164, 0.179, 0.204)
MOSSE	0.158	(0.151, 0.154, 0.188)
GOTURN	0.139	(0.138, 0.147, 0.162)

The first column presents the results of the proposed system in which each tracker is initialized with the bounding box given by HiC in its first valid HOI detection. The last column reports the SS, NPS, and GSR results achieved by each tracker with the OPE protocol on the sub-sequences yielded by the HOI labels. Best results, per measure, are highlighted in Bold, second-best in Bolditalic, third-best in Italic

### Contribution of Trackers to FPV Tasks

To understand if the employment of trackers brings advantages with respect to the more standard object localization solutions used in FPV (Damen et al., 2018; Shan et al., 2020), we compared the Recall results of the trackers presented in Table 6 with the Recall results of the original hand-object interaction detector HiC (Shan et al., 2020) which processes the frames independently. This solution achieves a Recall of 0.113 which results very low when compared to the 0.248, 0.246, and 0.245 achieved by the pipelines exploiting STARK, LTMU-H, TransT, respectively.

In addition, we compared the performance the EK-55-trained Faster R-CNN (Damen et al., 2018) and HiC (Shan et al., 2020) when used as pure object detectors (not exploiting temporal information for tracking as in the TbyD-F/H baselines). In this case, for Faster-R-CNN, at every frame, we consider as output the bounding box having the highest score associated to the category of the target object in the video, while for HiC we just take the object bounding box having the largest score (HiC provides class-agnostic object detections). On the sequences of TREK-150  the first solution achieves an OPE-based SS, NPS, and GSR of 0.323, 0.369, 0.044 respectively, and runs at 1 FPS, while the second reaches SS 0.411, NPS 0.438, GSR 0.007, at 8 FPS. Comparing these results with those of the TbyD-F/H baselines, we see the advantage of performing tracking, since all the metric scores are improved. Moreover, if we compare the detectors’ results with the ones presented in the overall study, we clearly notice that trackers, even when initialized by a detection module, can deliver faster, more accurate, and much temporally longer object localization than detectors.

Overall, these outcomes demonstrate that visual object trackers can bring benefits to FPV application pipelines. In addition to the ability of maintaining reference to specific object instances, the advantages of tracking are achieved in terms of better object localization and efficiency. We hence expect that trackers will likely gain more importance in FPV as new methodologies explicitly considering the first person point of view are investigated.

## Conclusions

In this paper, we proposed the first systematic evaluation of visual object tracking in first person vision (FPV). The analysis has been conducted with standard and novel measures on the newly introduced TREK-150 benchmark, which contains 150 video sequences extracted from the EK (Damen et al., 2018, 2021) FPV dataset. TREK-150 has been densely annotated with 97K bounding-boxes, 17 sequence attributes, 20 action verb attributes, and 34 target object attributes, as well as with 167K spatial annotations for the camera wearer’s hands and 78K states of interaction with the target object. The performance of 38 state-of-the-art generic object visual trackers and four baseline FPV trackers was analysed extensively on the proposed dataset. The investigation has conducted to the following conclusions. The performance of all the benchmarked trackers is decreased when compared with the respective accuracy on other popular visual object tracking benchmarks. This is explained by the different nature of images and the particular characteristics introduced by FPV which offer new and challenging conditions for the current knowledge in the visual tracking domain and the lack of tracking-specific FPV data. The analysis revealed that deep learning-based trackers employing online adaptation techniques achieve better performance than the trackers based on siamese neural networks or on handcrafted features. Among the different methodologies based on this approach, the transformer-based worked the best and hence is a promising future direction. This exploration could involve the curation of large-scale diverse tracking-specific data. The introduction of FPV-specific object localization modules, such as HOI models, in a tracking pipeline increased its performance, demonstrating that particular cues about the domain influence the tracking accuracy. These results highlighted the potential direction of joint hand-object tracking, and we expect successful methodologies to take into account also cues about the camera wearer’s surroundings. The performance of the trackers was then studied in relation to specific attributes characterising the visual appearance of the target and the scene. It turned out that the most challenging factors for trackers are the target’s out of view, its full occlusions, its low resolution, as well the presence of similar objects or of fast motion in the scene. Trackers were also analyzed based on the action performed by the camera wearer as well as the object category the target belongs to. It resulted that actions causing the change of state, shape, or aspect ratio of the target affected the trackers more than the actions causing only spatial changes. We think that trackers incorporating semantic information about the person’s action could be an interesting direction of investigation. We observed that rigid thin-shaped objects are among the hardest ones to track. Finally, we evaluated the trackers in the context of the FPV-specific application of video-based hand-object interaction detection. We included each tracker in a pipeline to tackle such a problem, and evaluated the performance of the system to quantify the tracker’s contribution. We observed that the trackers demonstrate a behavior that is consistent with their overall performance on the sequences of TREK-150. Even though FPV introduced challenging factors for trackers, the results in such a specific task demonstrated that current trackers can be used successfully if the video sequences in which tracking is required are not too long. We also demonstrated that trackers bring advantages in terms of object referral and localization, and efficiency, over object detection. We think that an effective and efficient integration of tracking methodologies with those of FPV downstream applications is a relevant problem to study. In conclusion, we believe that there is potential in improving FPV pipelines by employing visual trackers as well as there is room for the improvement of the performance of visual object trackers in this new domain.

## Supplementary Information

Below is the link to the electronic supplementary material.

Supplementary file 1 (pdf 7149 KB)

## Footnotes

1 Annotations, trackers’ results, and code are available at https://machinelearning.uniud.it/datasets/trek150/.
