# Supplementary material for: Visual Object Tracking in First Person Vision
Source: Int J Comput Vis. 2022 Oct 18;131(1):259–83. doi: 10.1007/s11263-022-01694-6 (PMC9816211; doi:10.1007/s11263-022-01694-6)
Supplement: Supplementary file 1 — (pdf 7149 KB) [file 11263_2022_1694_MOESM1_ESM.pdf]

"Visual Object Tracking in First Person Vision"

Supplementary Document

Matteo Dunnhofer, Antonino Furnari, Giovanni Maria Farinella, Christian Micheloni
Corresponding author e-mail: matteo.dunnhofer@uniud.it

8 The TREK-150 Benchmark

8.1 Further Motivations and Details

In this section, we provide further motivations and details behind the construction of the TREK-150 dataset.

Frame Rate

The video sequences included in TREK-150 have the frame rate of 60 FPS inherited from EK. According to the authors [19, 20], EK has been acquired with such a frame rate because of the proximity of the camera point of view and the main scene (i.e. manipulated objects) which causes very fast motion, and heavy motion blur due to the camera wearer movements (especially when he/she moves the head). We empirically checked the amount of fast motion by assessing the average normalized motion happening between the bounding boxes of consecutive frames that include such a condition. The motion has been quantified as the distance between the center of two consecutive ground-truth bounding boxes normalized by the frame size. Considering a subsampled version of TREK-150 at 30 FPS, such a value achieves 0.075. This is higher than the values present in other tracking benchmarks such as the 0.068 of OTB-100, the 0.033 of UAV123, or the 0.049 of the 30 FPS-version of NfS. These comparisons demonstrate that the FPV scenario effectively includes challenging conditions due to the faster motion of the targets/scene. Considering the original frame rate of 60 FPS, the fast motion quantity of TREK-150 is reduced to 0.062, which is comparable to the values obtained in other tracking benchmarks.

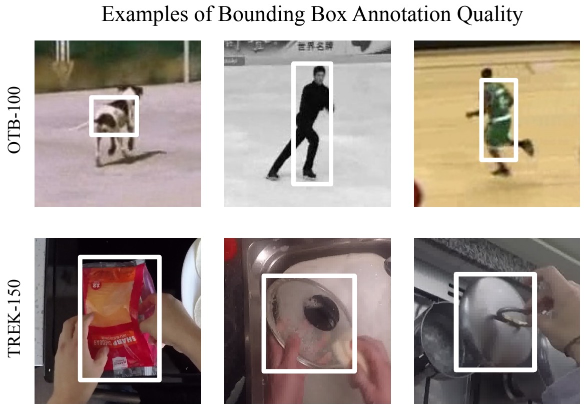

Fig. 13: Examples of the quality of the bounding box annotations contained in TREK-150 in comparison with the ones available in the popular OTB-100 benchmark. TREK-150 provides careful and high-quality annotations that tightly enclose all the target objects.

Sophisticated Target Representations

We would like to point out the difficulty that the FPV setting poses on the creation of more sophisticated annotations for the object categories appearing commonly in FPV scenarios. Figure 14 shows some examples of these. The first two images from the left show the objects "cheese" and "onion" (these are considered as single objects according to the EK-55 annotations [19]) which prevent the determination of the angle for an oriented bounding box, or an even accurate segmentation mask due to their spatial sparsity. The two images on the right present objects for which providing a segmentation is very ambiguous. Indeed, most of the pixels in the image area of the knife (third image) belong actually to foam, while the heavy motion blur happening on the object of the fourth image (where the target is a bottle) prevents the definition of the actual pixels belonging to the object. In all these scenarios, axis-aligned bounding boxes result in robust target representations that provide a consistent delineation of the object. Hence, to make the annotations consistent across the whole dataset, we employed such representations for TREK-150.

Sequence Annotations

To study the performance of trackers under different aspects, the sequences of TREK-150 have been associated with one or more of 17 attributes that indicate the visual variability of the target in the sequence (see Table 2 of the main paper for the details). The extended usage of this practice [34, 41, 48, 59, 77, 78, 108]

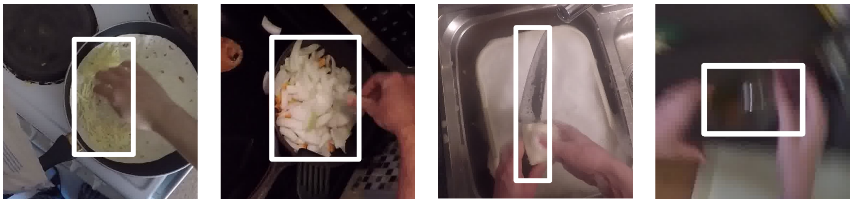

Fig. 14: Examples of target objects contained in TREK-150 that are difficult to represent with more sophisticated representations (e.g. rotated bounding box or segmentation mask). The first two images from the left show objects such as “cheese” and “onion” which prevent the determination of the angle for an oriented bounding-box, or an accurate segmentation mask. The last two images present objects which prevent a consistent definition of a segmentation mask.

showed how this kind of labeling is sufficient to estimate the trackers’ performance on particular scenarios. We therefore followed such an approach to associate labels on TREK-150’s videos. However, we argue that, by using this labeling setting, attention must be paid to how trackers are evaluated. The standard OPE protocol, which has been generally used to perform such evaluations, could lead to less accurate estimates. For example, it could happen that a tracker would fail for some event described by an attribute (e.g. FOC) in the first frames of a video, but that the sequence also contains some other event (e.g. MB) in the end. With the score averaging procedure defined by the OPE protocol, the low results achieved due to the first event would set low scores also for the second event, while the tracker failed just for the first one. Therefore, the performance estimate for the second attribute would not be realistic. We believe that a reasonable option is to use a more robust evaluation protocol such as the multi-start evaluation (MSE). Thanks to its points of initialization which generate multiple diverse sub-sequences, this protocol allows a tracker to better cover all the possible situations happening along the videos, both forward and backward in time. All the results achieved on the sub-sequences are then averaged to obtain the overall scores on a sequence. We think the scores computed in this way to be more robust and accurate estimates of the real performance of the trackers. Hence, in this work, we follow such an approach to evaluate trackers over sequence attributes.

Even though we provide per-frame labels to describe the interaction happening between the camera wearer and the target object (LHI, RHI, BHI labels), we considered the sequence attributes 1H and 2H in order to have a direct and more consistent assessment of the impact of the conditions they indicate in relation to the other attributes associated to sequences.

Differences With Other Tracking Benchmarks

We believe that the proposed TREK-150 benchmark dataset offers *complementary* features with respect to the existing visual tracking benchmarks.

Table 1 and Figure 2(a-b) of the main paper show that TREK-150 provides complementary characteristics to what is available today to study the performance of visual trackers. Particularly, our proposed dataset offers different distributions of the common challenging factors encountered in other datasets. For example, TREK-150 includes a larger number of examples with occlusions (POC), fast motion (FM), scale change (SC), aspect ratio change (ARC), illumination variation (IV), and motion blur (MB), while it provides a competitive number of scenarios for low resolution (LR), full occlusion (FOC), deformable objects (DEF), and presence of similar objects (SOB). Additionally, even though the 4 new attributes high resolution (HR), head motion (HM), one-hand interaction (1H), two-hands interaction (2H), define particular FPV scenarios, we think that they can be of interest even for the visual tracking community. For example, as shown by the second row of images of Figure 13, 1H and 2H can be considered as attributes that define different levels of occlusion, as objects manipulated with two hands generally cause more extended hiding of the targets. Besides these sequence-level features, TREK-150 offers up to 34 target categories which, to the best of our knowledge, have never been studied. As shown by the Figures 13 and 14, these objects have challenging appearances (e.g. transparent or reflective objects like lids, bottles, or food boxes) and shapes (e.g. knives,

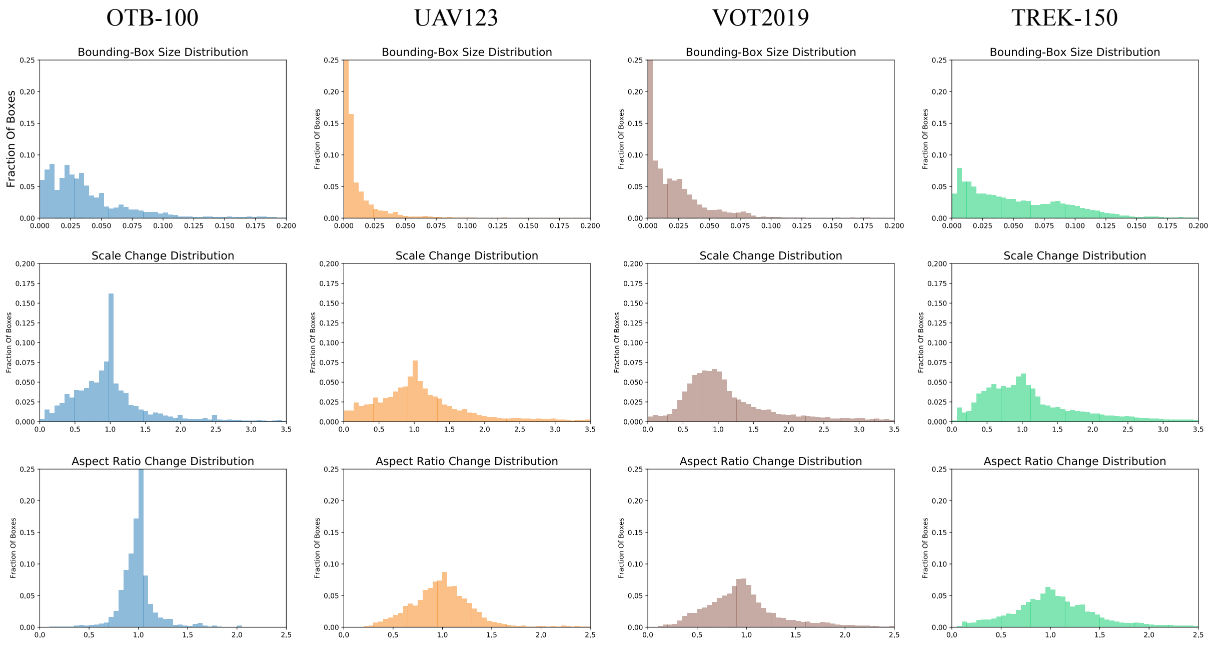

Fig. 15: Comparison between TREK-150 (last column of plots) and other popular visual tracking benchmarks on the distributions computed for different bounding box characteristics. Each column of plots reports the distribution of bounding box sizes, scale changes, and aspect ratio change (the x-axis of each plot reports the range of the bounding box statistic).

spoons, cut food) that change dramatically due to the interaction or motion induced by the camera wearer.

We additionally computed some statistics on the bounding box annotations for the targets in TREK-150 and on those of other popular visual object tracking benchmarks to understand whether FPV offers different motions of the objects. As highlighted by Figure 15, our dataset exhibits different distributions and thus offers different behaviors of the target appearances and motions. Observing the top plot of the last column, it can be noted that TREK-150 has a wider distribution of bounding box dimensions, making it suitable for the evaluation of trackers with targets of many different sizes. Particularly, TREK-150 has a larger number of bounding boxes with greater dimension. The plot just below shows that TREK-150 provides more annotations to assess the capabilities in the tracking of objects that become smaller. Finally, the last plot shows a wider distribution for the aspect ratio change, telling that TREK-150 offers a large variety of examples to evaluate the capabilities of trackers in predicting the shape change of objects.

Additionally to these characteristics, we think TREK-150 is interesting because it allows the study of visual object tracking in unconstrained scenarios of *every-day* situations.

9 Trackers

9.1 Details of the TbyD-F/H Baselines

In this section, we provide the details of the other TbyD-F/H versions whose results are presented in Table 4. For the baseline that uses SORT [8] (whose results are given in rows 1 and 4 of Table 4), we applied such bounding box association method over the detections of the underlying detector. Specifically, at the first frame we initialize a Kalman filter for the initial target object's bounding box and assign the ID predicted by this filter as 0. Then, at every subsequent frame, we first obtain all the object detections by inputting the detector with the frame. Such localizations are paired with the respective predicted confidence scores and are given as input to SORT. The latter associates the new detections with the memorized tracklets and, through Kalman filters, refines the localizations for all the objects observed in the scene. Since our problem requires the localization of a single target object (SOT),

we return the bounding box associated to ID 0 as the output of this TbyD-F/H version.

The baseline whose results are given in rows 3 and 6 of Table 4 uses a combination of the strategy based on the previously predicted bounding box and SORT [8]. In more detail, at the first frame of a sequence, we initialize a Kalman filter with the bounding box for the target of interest and assign the ID as 0. The given bounding box is also memorized as the previous target position. At every other frame, we first run the detector and among all the given detections, we retain the one having the largest IoU with the memorized bounding box. This bounding box is paired with the respective score given by the detector, and the resulting concatenated vector is inputted to SORT which then provides the refined bounding box for the target. If the detector does not provide detections or no box has an IoU greater than zero with the memorized box, the latter is used as output localization and SORT is inputted with an empty set of detections/scores.

10 Evaluation

In this section, we describe the additional evaluation protocols and metrics used in our study.

Real-Time Evaluation

Since many FPV tasks such as object interaction [21] and early action recognition [40], or action anticipation [19], require real-time computation, we evaluate trackers in such a setting by following the details given in [55, 62]. This protocol, which we refer to as RTE, is similar to OPE. A tracker is initialized with the ground-truth in the first frame of a sequence. Then the algorithm is presented with a new frame only after its execution over the previous frame has finished. The new presented frame is the last frame available for the time instant in which the tracker becomes ready to be executed, considering that frames occur regularly based on the frame rate of the video. In other words, all the frames occurring in the time interval between the start and end time instants of the tracker's execution are skipped. For all such frames, the last box given by the tracker is used as location for the target. The overall performance scores (SS, NPS, GSR) are ultimately obtained as for the OPE protocol. Together with those values, we evaluate the trackers' processing speed in frames per second (FPS) to quantify their efficiency.

11 Results

In this section, we provide complete results for all the set of 42 trackers analyzed in this study. We also report on the real-time performance of trackers and give additional insights on some of the deep learning-based methodologies considered in our work.

11.1 Complete Results

In Figure 16, the OPE-based SS, NPS, GSR plots and scores are given for all the 42 considered trackers. Figure 17 reports the MSE-based scores, while Figures 21, 22, 23 show the MSE-based scores with respect to attributes, verbs, and nouns respectively. Figure 19 includes the change in SS, NPS, GSR after the trackers' initialization with object detectors [19, 93], while Tables 7 and 8 report the results of the HOI evaluation with the HiC-based pipeline (Table 7) and with the oracle-based pipeline (Table 8).

11.2 Processing Speed Study

Table 9 reports the FPS performance of the 42 trackers and the SS, NPS, and GSR scores achieved under the RTE protocol. None of the trackers achieve the frame rate speed of 60 FPS. We argue that this is due to the full HD resolution of frames which requires demanding image crop and resize operations with targets of considerable size. Taking into consideration the tracking approaches, we observe that trackers based on single-shot siamese networks (e.g. Ocean, SiamBAN, SiamRPN++) or on light online adaptation techniques such as the target template change (e.g. as performed by TransT, STARK, STMTrack) emerge as the fastest trackers and exhibit a less significant performance drop of the proposed scores. In particular, the decrease in SS of SiamBAN and SiamRPN++ is of 3.7% and 4.7%, respectively, with respect to the OPE results. TransT and STARK exhibit a larger performance drop of 5.3 % and 6.1% respectively, but their robustness makes their overall real-time performance much higher than SiamBAN and SiamRPN++. Due to the reliance on heavier online learning mechanisms, trackers like KeepTrack, PrDiMP, ATOM, KYS, ECO achieve a lower processing speed that consequently causes a major accuracy loss in real-time scenarios. The proposed FPV tracking baselines show a consistent drop in real-time settings. This is due to their reliance on demanding models such as the FPV object detectors [19, 93]. LTMU-F/H result a bit better on this point since they do not execute the detector at every frame,

Table 7: Results of the experiment in which trackers are evaluated by the Recall of an FPV HOI detection pipeline where trackers are used as localization method for the object involved in the interaction. The first column presents the results of the proposed system in which each tracker is initialized with the bounding box given by HiC in its first valid HOI detection. The last column reports the SS, NPS, and GSR results achieved by each tracker with the OPE protocol on the subsequences yielded by the HOI labels. Best results, per measure, are highlighted in **gold**, second-best in **silver**, third-best in **bronze**.

Tracker	Recall	(SS, NPS, GSR)
STARK	0.248	(0.211, 0.221, 0.222)
LTMU-H	0.246	(0.210, 0.222, 0.217)
LTMU-F	0.245	(0.210, 0.221, 0.216)
TransT	0.243	(0.208, 0.221, 0.229)
LTMU	0.241	(0.204, 0.220, 0.208)
TrDiMP	0.239	(0.205, 0.213, 0.222)
TbyD-H	0.238	(0.205, 0.223, 0.163)
LightTrack	0.233	(0.197, 0.212, 0.228)
KeepTrack	0.232	(0.201, 0.214, 0.212)
PrDiMP	0.228	(0.194, 0.207, 0.206)
SiamRPN++	0.227	(0.191, 0.206, 0.209)
SiamBAN	0.226	(0.192, 0.209, 0.209)
TbyD-F	0.220	(0.184, 0.202, 0.179)
SiamMask	0.219	(0.185, 0.198, 0.213)
Ocean	0.218	(0.189, 0.203, 0.207)
STMTrack	0.216	(0.196, 0.202, 0.219)
KYS	0.212	(0.187, 0.199, 0.218)
D3S	0.211	(0.187, 0.199, 0.208)
ECO	0.211	(0.181, 0.196, 0.217)
DiMP	0.210	(0.186, 0.198, 0.211)
SiamDW	0.209	(0.179, 0.198, 0.208)
MetaCrest	0.209	(0.178, 0.188, 0.212)
TRASFUST	0.208	(0.188, 0.199, 0.210)
ATOM	0.207	(0.186, 0.198, 0.213)
VITAL	0.198	(0.178, 0.192, 0.213)
SiamFC++	0.198	(0.173, 0.185, 0.184)
SiamFC	0.195	(0.171, 0.180, 0.195)
GlobalTrack	0.195	(0.170, 0.180, 0.144)
BACF	0.188	(0.170, 0.189, 0.206)
MDNet	0.185	(0.170, 0.185, 0.207)
DSLT	0.185	(0.165, 0.179, 0.188)
Staple	0.182	(0.164, 0.179, 0.204)
SiamGAT	0.182	(0.162, 0.167, 0.181)
UpdateNet	0.182	(0.158, 0.158, 0.185)
MCCTH	0.179	(0.165, 0.172, 0.199)
DCFNet	0.179	(0.164, 0.171, 0.200)
DSST	0.179	(0.163, 0.167, 0.203)
SPLT	0.178	(0.155, 0.168, 0.166)
STRCF	0.169	(0.161, 0.178, 0.193)
KCF	0.166	(0.154, 0.159, 0.190)
MOSSE	0.158	(0.151, 0.154, 0.188)
GOTURN	0.139	(0.138, 0.147, 0.162)

Table 8: Results of the experiment in which trackers are evaluated by the Recall of an FPV HOI detection pipeline where trackers are used as localization method for the object involved in the interaction. The first column presents the results of the proposed system in which each tracker is initialized with the ground-truth bounding box for the first frame labeled with the HOI. The last column reports the SS, NPS, and GSR results achieved by each tracker with the OPE protocol on the sub-sequences yielded by the HOI labels. Best results, per measure, are highlighted in **gold**, second-best in **silver**, third-best in **bronze**.

Tracker	Recall	(SS, NPS, GSR)
LTMU-H	0.754	(0.648, 0.680, 0.666)
STARK	0.750	(0.646, 0.677, 0.695)
LTMU-F	0.746	(0.641, 0.672, 0.667)
TransT	0.725	(0.631, 0.659, 0.708)
STMTrack	0.671	(0.595, 0.610, 0.726)
LightTrack	0.670	(0.586, 0.611, 0.710)
TrDiMP	0.669	(0.590, 0.606, 0.688)
LTMU	0.663	(0.578, 0.613, 0.623)
KeepTrack	0.661	(0.587, 0.611, 0.672)
SiamRPN++	0.660	(0.577, 0.605, 0.678)
PrDiMP	0.653	(0.573, 0.600, 0.669)
SiamBAN	0.637	(0.566, 0.603, 0.666)
TRASFUST	0.617	(0.562, 0.594, 0.659)
TbyD-F	0.617	(0.528, 0.578, 0.509)
SiamMask	0.615	(0.542, 0.576, 0.643)
ATOM	0.614	(0.559, 0.590, 0.670)
ECO	0.613	(0.556, 0.600, 0.701)
VITAL	0.610	(0.553, 0.580, 0.682)
KYS	0.609	(0.559, 0.575, 0.660)
Ocean	0.608	(0.538, 0.560, 0.649)
DiMP	0.607	(0.547, 0.574, 0.650)
TbyD-H	0.603	(0.544, 0.582, 0.389)
D3S	0.598	(0.541, 0.566, 0.638)
SiamDW	0.572	(0.512, 0.552, 0.642)
MetaCrest	0.569	(0.518, 0.535, 0.662)
MDNet	0.570	(0.518, 0.560, 0.664)
GlobalTrack	0.563	(0.493, 0.507, 0.440)
SiamFC	0.549	(0.504, 0.530, 0.617)
MCCTH	0.547	(0.510, 0.529, 0.650)
Staple	0.544	(0.510, 0.537, 0.655)
SiamFC++	0.542	(0.495, 0.519, 0.602)
UpdateNet	0.540	(0.470, 0.473, 0.612)
BACF	0.536	(0.507, 0.551, 0.665)
DCFNet	0.531	(0.508, 0.523, 0.651)
DSST	0.531	(0.503, 0.518, 0.653)
DSLT	0.514	(0.481, 0.507, 0.587)
KCF	0.488	(0.472, 0.482, 0.622)
SiamGAT	0.487	(0.465, 0.468, 0.533)
STRCF	0.481	(0.466, 0.501, 0.612)
SPLT	0.480	(0.450, 0.473, 0.541)
MOSSE	0.459	(0.459, 0.459, 0.614)
GOTURN	0.367	(0.384, 0.404, 0.509)

but only when triggered by the target verification mechanism. The performance drop is particularly large with respect the underlying tracker STARK because of the long initialization time (that lasts 8 seconds on average) that LTMU-F/H takes to create all the models involved in the pipeline. TbyD-F/H execute the time-consuming detectors at every frame, resulting in an even lower performance due to the many frames skipped during the tracking.

In general, we observe that the GSR score is the measure on which all trackers present the major performance drop in the real-time setting, suggesting that particular effort should be spent to make trackers better address longer references to objects in real-time scenarios. Overall, we can say that trackers like TransT

Table 9: Performance achieved by the 42 trackers benchmarked on TREK-150 using the RTE protocol. Best results, per measure, are highlighted in gold, second-best in silver, third-best in bronze.

Tracker	FPS	SS	NPS	GSR
TransT	19	0.462	0.471	0.394
STARK	14	0.453	0.456	0.345
STMTrack	13	0.434	0.440	0.407
TrDiMP	9	0.389	0.378	0.287
LightTrack	8	0.376	0.373	0.335
Ocean	21	0.365	0.358	0.294
SiamRPN++	23	0.362	0.356	0.293
SiamBAN	24	0.360	0.369	0.313
PrDiMP	13	0.352	0.349	0.243
KeepTrack	9	0.345	0.335	0.188
DiMP	16	0.336	0.331	0.224
SiamMask	23	0.335	0.333	0.298
SiamFC++	45	0.330	0.331	0.308
SiamDW	32	0.327	0.334	0.317
KYS	12	0.327	0.317	0.219
ATOM	15	0.319	0.312	0.179
SiamGAT	20	0.314	0.306	0.257
UpdateNet	21	0.311	0.297	0.295
DCFNet	49	0.299	0.286	0.335
TRASFUST	13	0.296	0.270	0.185
SiamFC	34	0.293	0.295	0.280
D3S	16	0.276	0.263	0.182
BACF	9	0.276	0.262	0.234
SPLT	8	0.265	0.247	0.203
STRCF	10	0.264	0.250	0.218
DSLT	7	0.260	0.234	0.211
ECO	15	0.252	0.231	0.173
GlobalTrack	8	0.253	0.227	0.139
MCCTH	8	0.251	0.231	0.232
Staple	13	0.249	0.236	0.234
GOTURN	44	0.247	0.242	0.119
LTMU-H	9	0.243	0.205	0.163
MOSSE	26	0.227	0.190	0.244
LTMU-F	7	0.222	0.180	0.162
LTMU	3	0.213	0.178	0.161
MetaCrest	8	0.207	0.175	0.165
VITAL	4	0.204	0.165	0.158
DSST	2	0.191	0.145	0.161
TbyD-F	1	0.191	0.135	0.163
KCF	6	0.186	0.157	0.177
MDNet	1	0.185	0.140	0.161
TbyD-H	8	0.175	0.140	0.127

and STARK are currently the most suitable methods to employ for the development of real-time FPV applications requiring object tracking. Given their limited performance decrease between the OPE and RTE results, siamese-based trackers could serve as promising alternatives if their tracking accuracy and robustness are improved.

11.3 Study of Deep Learning Trackers

Considering that nowadays most of the state-of-the-art solutions are based on deep neural networks, in this section we provide insights about how the FPV performance of such methods depends on the training data and on the neural network design. In these experiments, we consider the trackers SiamRPN++, DiMP, and STARK, as the representative methods of the popular and successful approaches of siamese networks, deep discriminative correlation filters, and transformers.

Table 10: OPE-based performance of the deep learning-based trackers SiamRPN++, DiMP, STARK on TREK-150 after being trained separately on each of the most popular generic object TPV datasets (TrackingNet, GOT-10k, LaSOT).

Tracker	Metric	Training Dataset TrackingNet	GOT-10k	LaSOT
SiamRPN++	SS	0.307	0.332	0.281
	NPS	0.315	0.343	0.290
	GSR	0.298	0.318	0.280
DiMP	SS	0.349	0.322	0.378
	NPS	0.349	0.327	0.396
	GSR	0.331	0.291	0.343
STARK	SS	0.314	0.315	0.340
	NPS	0.314	0.304	0.332
	GSR	0.244	0.182	0.199

Impact of Training Data

As reported in Table 3, deep learning-based trackers use several different datasets to optimize the neural network-based modules used inside their processing pipelines. At a first glance, it is not easy to correlate the tracking performance in FPV with the training set reported in such a table. To overcome this problem, we re-trained the 3 representative trackers SiamRPN++, DiMP, STARK, on the training set of each of the most popular large-scale tracking datasets currently available, i.e. TrackingNet, GOT-10k, and LaSOT. These datasets comprise videos of generic objects acquired from a third person view (TPV) generally. Despite they provide similar characteristics in the video perspective and target's nature, such datasets have different features, especially in the size and the object categories. For example, TrackingNet offers a training set of more than 30K videos and 14M frames with 21 target object categories, GOT-10k provides 9.3K videos for a total of 1.4M frames and 480 object categories, while LaSOT's training set comprises 1.1K videos, 2.8M frames, and 70 object categories. To train the trackers, we followed the instructions given in the original papers and code repositories (which were publicly available). We kept the same hyperparameters for all the trackers and swapped only the training dataset. For the STARK tracker, we modified only the number of training epochs to a total of 20 (for both the two training stages) in order to achieve a reasonable training time on our hardware. We found such a number

Table 11: Performance of the deep learning-based trackers SiamRPN++, DiMP, STARK on TREK-150 when executed with different CNN backbones. The trackers have been executed under the OPE protocol.

Tracker	Metric	Backbone CNN AlexNet	ResNet-18	ResNet-50	ResNet-101
SiamRPN++	SS	0.345	-	0.380	-
	NPS	0.353	-	0.389	-
	GSR	0.325	-	0.350	-
DiMP	SS	-	0.373	0.386	-
	NPS	-	0.383	0.392	-
	GSR	-	0.339	0.356	-
STARK	SS	-	-	0.492	0.490
	NPS	-	-	0.504	0.505
	GSR	-	-	0.395	0.389

Table 12: Performance of the deep learning trackers SiamRPN++, DiMP, STARK on TREK-150 when trained on datasets of generic object tracking (TPV) and on a large-scale dataset for FPV object detection.

Tracker	Training Data	OPE SS	OPE NPS	OPE GSR	MSE SS	MSE NPS	MSE GSR
SiamRPN++	TPV	0.380	0.389	0.350	0.408	0.427	0.352
	FPV	0.343	0.364	0.327	0.376	0.402	0.322
DiMP	TPV	0.386	0.392	0.357	0.414	0.434	0.362
	FPV	0.418	0.441	0.382	0.454	0.480	0.394
STARK	TPV	0.340	0.332	0.199	0.373	0.366	0.209
	FPV	0.264	0.251	0.170	0.332	0.351	0.225

of epochs to be sufficient for achieving a significant tracking performance allowing the emergence of the performance difference after training on the different data distributions. The performance achieved by such trackers on TREK-150 after being trained with the aforementioned training sets is reported in Table 10. The relevant thing to understand here is how, for every methodology, the performance changes as the training set is changed. Siamese network-based trackers (SiamRPN++-like) benefit the most from the GOT-10k training set, suggesting that the large amount of object categories available in this dataset helps in making the tracking better generalize to the objects and scenarios present in FPV. Deep discriminative (DiMP-like) and transformer trackers (STARK-like) instead benefit the most from the LaSOT training set, suggesting that these methodologies need a balance between the number of object categories and the amount of appearance change in time (LaSOT provides long videos in which targets are subject to severe appearance changes) to perform well in FPV. Overall, we can observe the highest FPV tracking performance is not achieved with the largest number of videos and frames as available in TrackingNet. These results demonstrate that a large number of videos/frames is not sufficient to perform the best in FPV, but a more carefully designed training set should be used instead.

Impact of the CNN Backbone

Table 11 reports the performance of SiamRPN++, DiMP, and STARK trackers configured with different backbone networks. In this experiment, we wanted to understand which is the CNN that produces the best appearance features for FPV-based tracking. We used the pre-trained models provided by the respective authors and trained using the popular TPV tracking benchmarks. As can be easily noticed, for all the methodologies, the ResNet-50 is the backbone CNN that leads to the highest tracking performance in FPV.

Deep Learning Trackers Trained for FPV

We also assessed the impact of FPV-specific data to train SiamRPN++, DiMP, and STARK. At the time of writing, the only available data to be exploited for tracking is the subset of EK-55 frames labeled with the bounding boxes of objects for object detection tasks [19]. As expressed in Table 3, object detection datasets such as COCO [65] are exploited for training the deep models used inside trackers. The idea is to create synthetic videos from still images by applying transformations such as shift, scale, rotation, color jitter, to images of objects. We followed the same approach with EK-55's object detection images and annotations. As training set for the 3 trackers, we considered the subset of images associated to the camera wearers that are not present in TREK-150 (15 people). In total, 197229 frames and 214878 bounding boxes are present in this set for an overall number of 295 object categories. For comparison, COCO's training set has 118287 frames, 860001 bounding boxes belonging to 80 different categories. We retrained each of the trackers on such FPV training set by following the original instructions and hyperparameters. As for the earlier experiment, STARK was trained for 20 epochs in each of the two stages. In Table 12 we present a comparison between the trackers' instances trained for generic object tracking on standard TPV tracking datasets and those trained for FPV (for STARK we consider the best instance presented in Table 10 since it was trained with the same hyperparameters). The DiMP tracker is the only one benefiting from FPV-specific data for training. Indeed, its performance is

Table 13: Performance of the offline trackers SiamFC and SiamRPN++ on a subset of 50 sequences of TREK-150 without and with fine-tuning on the remaining 100 videos.

Tracker	Fine-tuning	OPE SS	OPE NPS	OPE GSR	MSE SS	MSE NPS	MSE GSR
SiamFC		0.311	0.332	0.317	0.307	0.317	0.307
	✓	0.267	0.275	0.278	0.287	0.305	0.292
SiamRPN++		0.384	0.395	0.377	0.367	0.385	0.333
	✓	0.348	0.407	0.313	0.336	0.406	0.314

improved by a good margin according to all the metrics. The other trackers, SiamRPN++ and STARK, do not benefit from this data and their tracking performance is weaker than the counterparts trained in TPV. These results suggest that online adaptative methods such as deep discriminative trackers benefit from an initial offline learning stage specifically designed for the domain of application. Less target adaptive trackers such as siamese networks and transformers require large-scale and diverse samples to learn an effective similarity function. Despite the improvement, the FPV-trained DiMP still does not achieve the performance that is observed in domains represented by other benchmarks (e.g. OTB-100, UAV123, LaSOT).

Deep Learning Trackers Fine-Tuned for FPV

The previous experiment revealed that large-scale FPV object detection data does not help those trackers based on siamese neural networks (e.g. SiamFC, SiamRPN++, SiamBAN, SiamGAT). A reason for this could be the fact that even though different transformations are applied to the still images, the appearance of the target does not change so severely as could be in the case of real object manipulation. Hence, we performed additional experiments on such methods to understand if their behavior can be improved by learning through object appearances that change through time. Our TREK-150 dataset is designed to evaluate the progress of visual tracking solutions in FPV and does not provide a large-scale database of learning examples as needed by these methods (a large-scale dataset for the training of FPV-specific trackers is out of the scope of this paper). In this view, TREK-150 well aligns with real-world datasets where millions of frames are not available for training. In such scenarios, the reasonable options the machine learning community suggests are to use the deep learning models as they are because of their general knowledge, or to adapt them through fine-tuning using a smaller training set. We carried out the second strategy by randomly splitting TREK-150 into a training and a test set of 100 and 50 videos respectively. We fine-tuned the popular offline trackers SiamFC and SiamRPN++ on such training set according to their original learning strategy. We then tested the fine-tuned versions on the test set and the results are reported in Table 13 in comparison with the original counterparts. The results show that the simple fine-tuning leads to substantial overfitting that cause the performance to drop in general. The exceptions are given by SiamRPN++'s NPS results which are increased through such adaptation procedure.

Overall, by considering the outcomes of this experiment and the ones of the previous assessment, as well as the results achieved by trackers trained for generic object tracking, we can conclude that FPV introduces challenging scenarios for tracking methods that cannot be completely addressed by the current availability of tracking data (both TPV and FPV) as well as the knowledge in deep learning-based visual tracking.

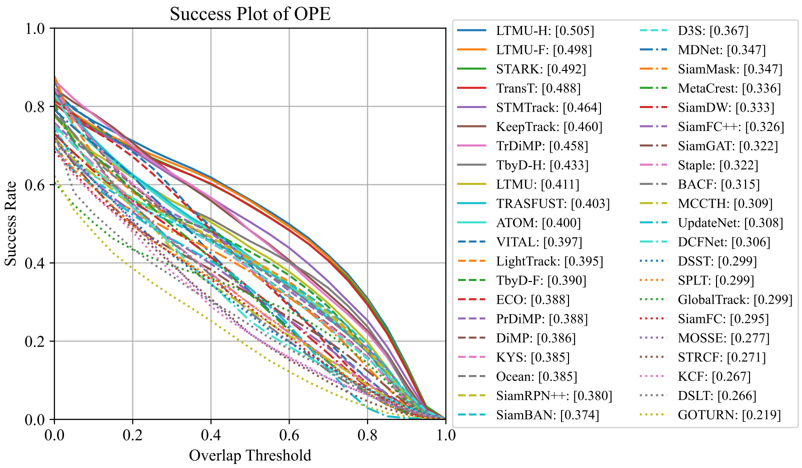

(a)

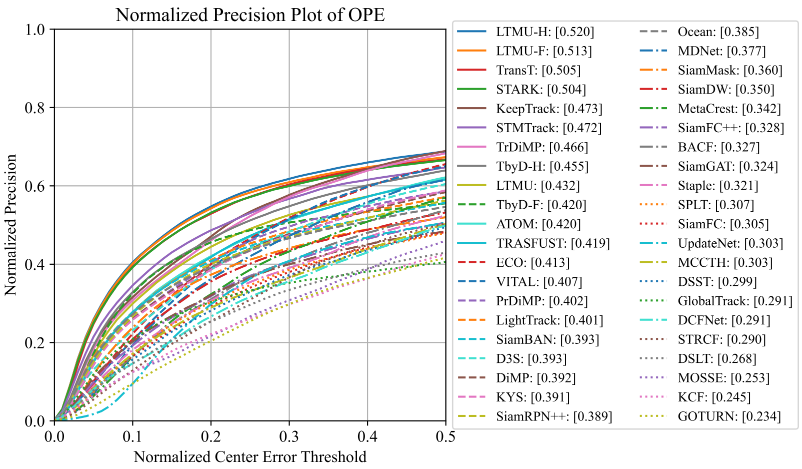

(b)

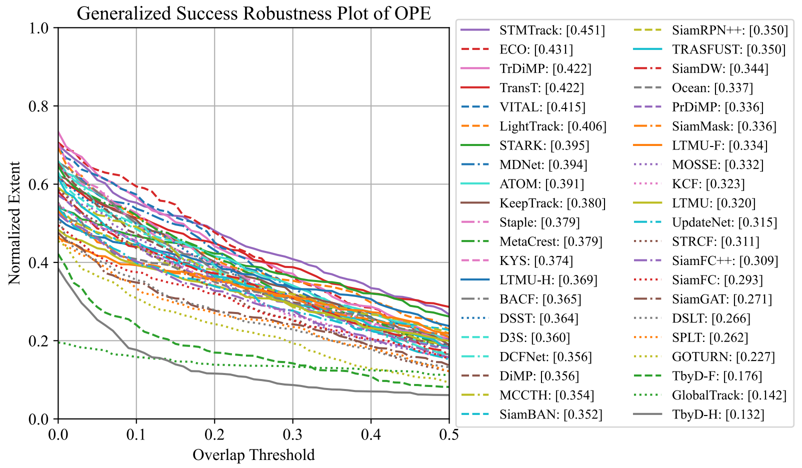

(c)

Fig. 16: Performance of all the 42 selected trackers on the proposed TREK-150 benchmark under the OPE protocol. In brackets, next to the trackers' names, we report the SS, NPS, and GSR values.

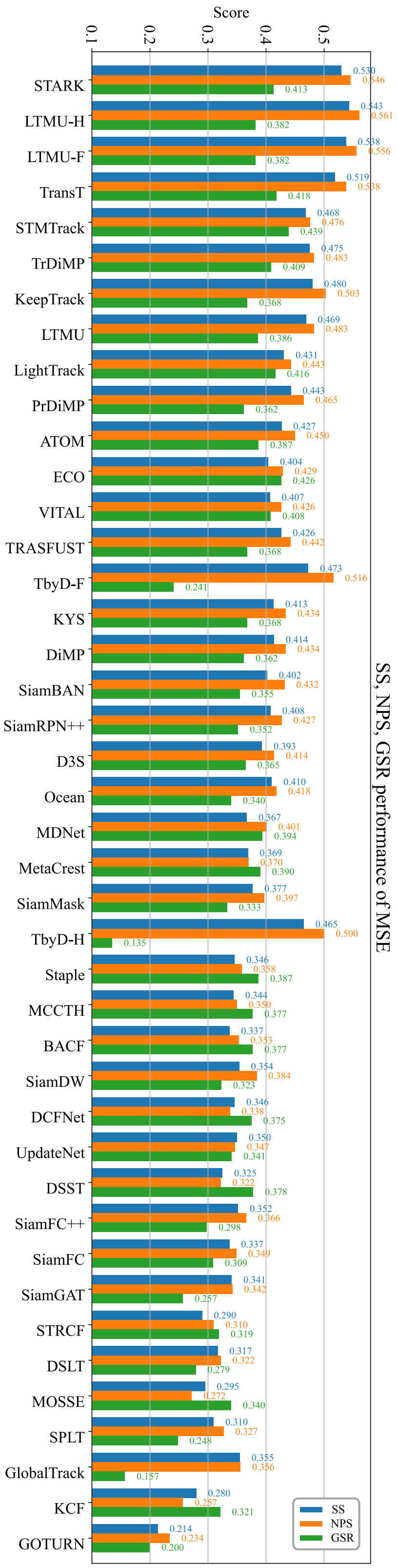

Fig. 17: SS, NPS, and GSR performance of the 42 benchmarked generic object trackers on the proposed TREK-150 benchmark achieved under the MSE protocol. The trackers are ordered by the average value of their SS, NPS, GSR scores.

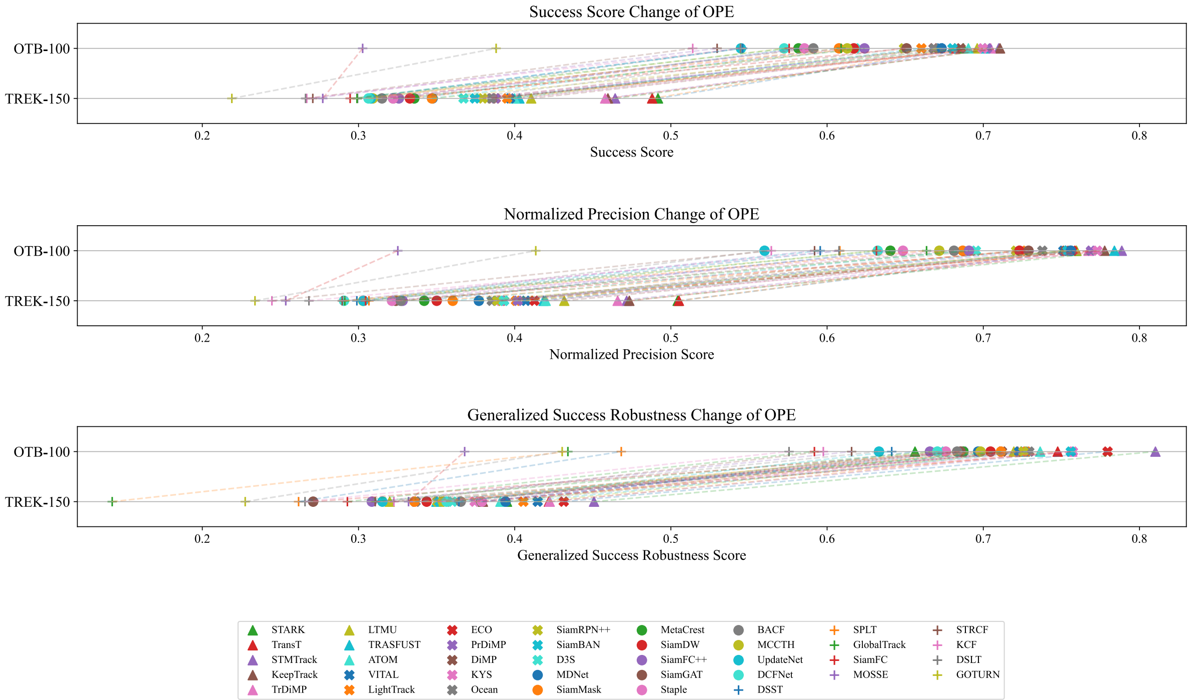

Fig. 18: Performance comparison of SS, NPS, and GSR scores obtained by the 38 benchmarked generic object trackers on the popular OTB-100 benchmark [108] and on the proposed TREK-150 benchmark under the OPE protocol.

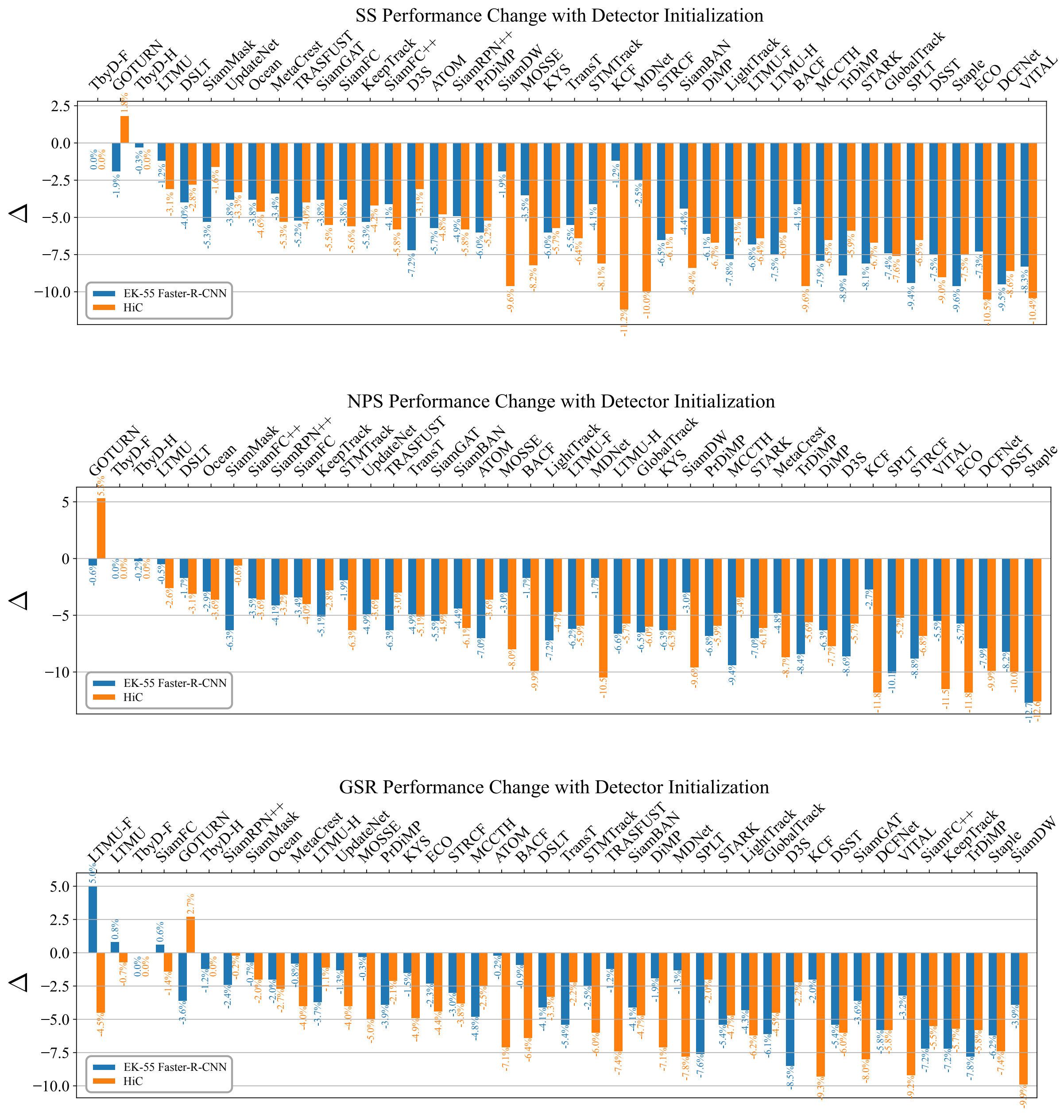

Fig. 19: Results of the OPE-D experiment in which the bounding box for initialization is given either by the EK-55 trained Faster-R-CNN [19] or the HiC detector [93]. The performance change with respect with the ground-truth initialization is reported for the SS, NPS, and GSR metrics. In each plot, the trackers are ordered by the average performance change of the two initialization experiments.

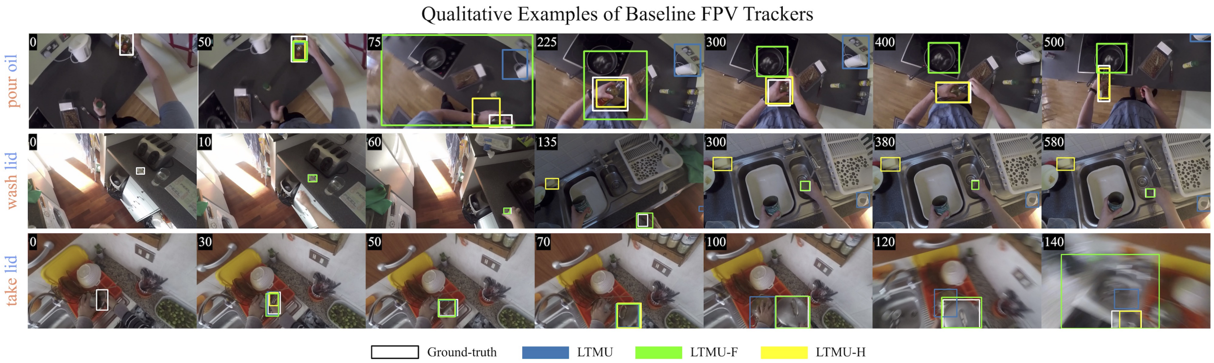

Fig. 20: Qualitative results of the baseline FPV trackers LTMU-F and LTMU-H in comparison with LTMU.

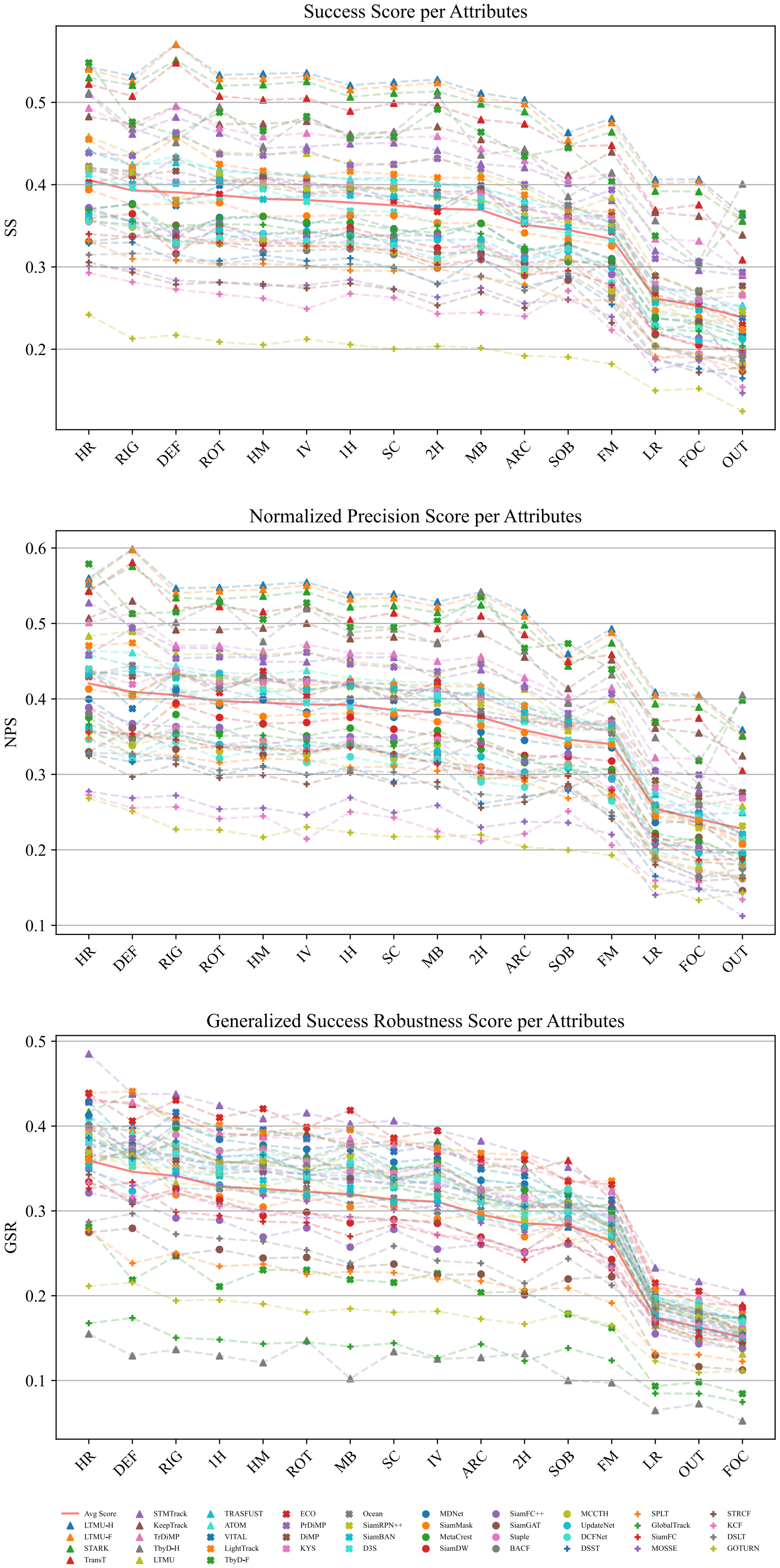

Fig. 21: SS, NPS, and GSR performance achieved under the MSE protocol of the 42 selected trackers with respect to the sequence attributes available in TREK-150. (The results for the POC attribute are not reported because this attribute is present in every sequence). The red plain line highlights the average tracker performance.

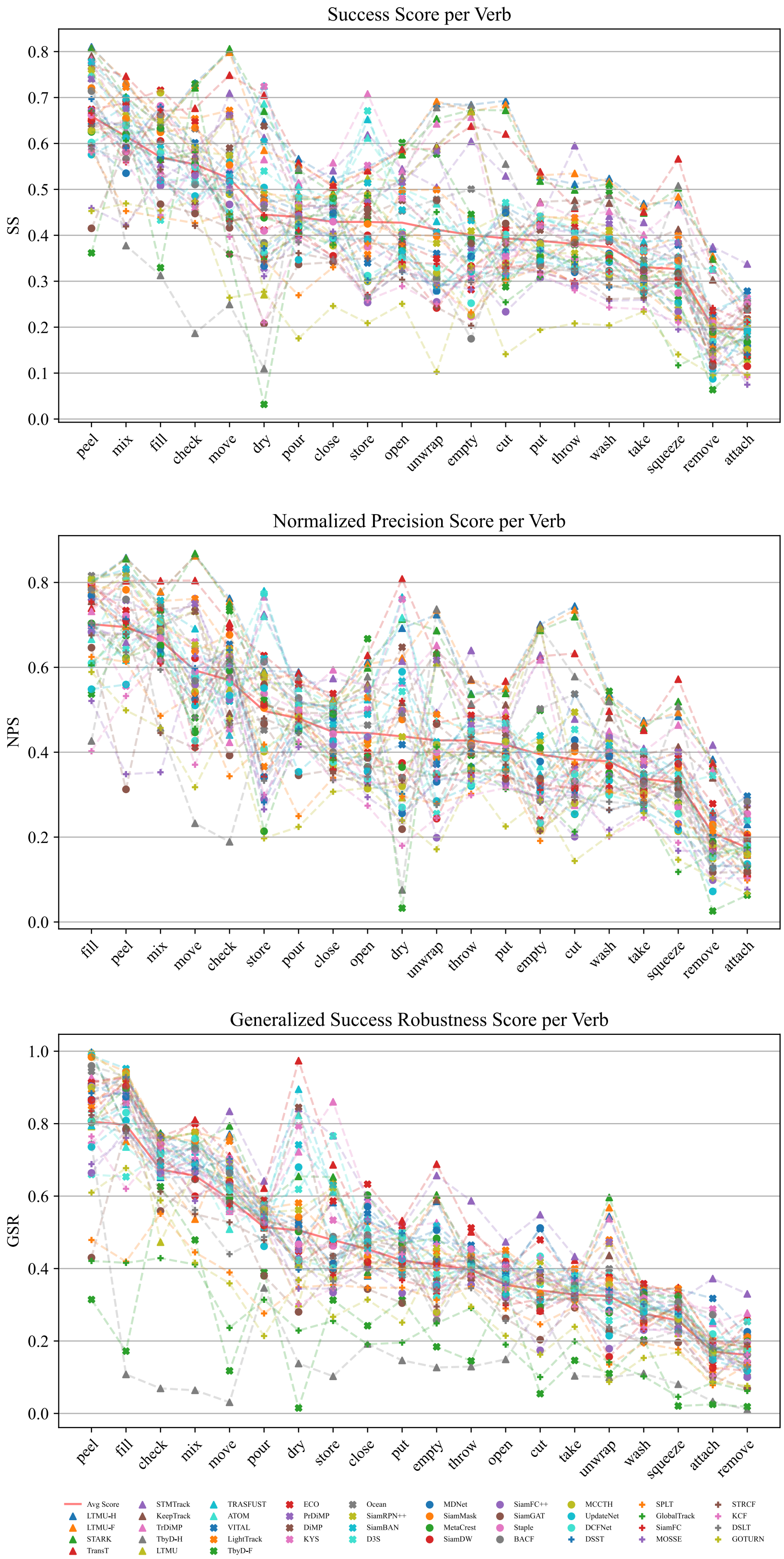

Fig. 22: SS, NPS, and GSR performance achieved under the MSE protocol of the 42 selected trackers with respect to the sequence verbs available in TREK-150. The red plain line highlights the average tracker performance.

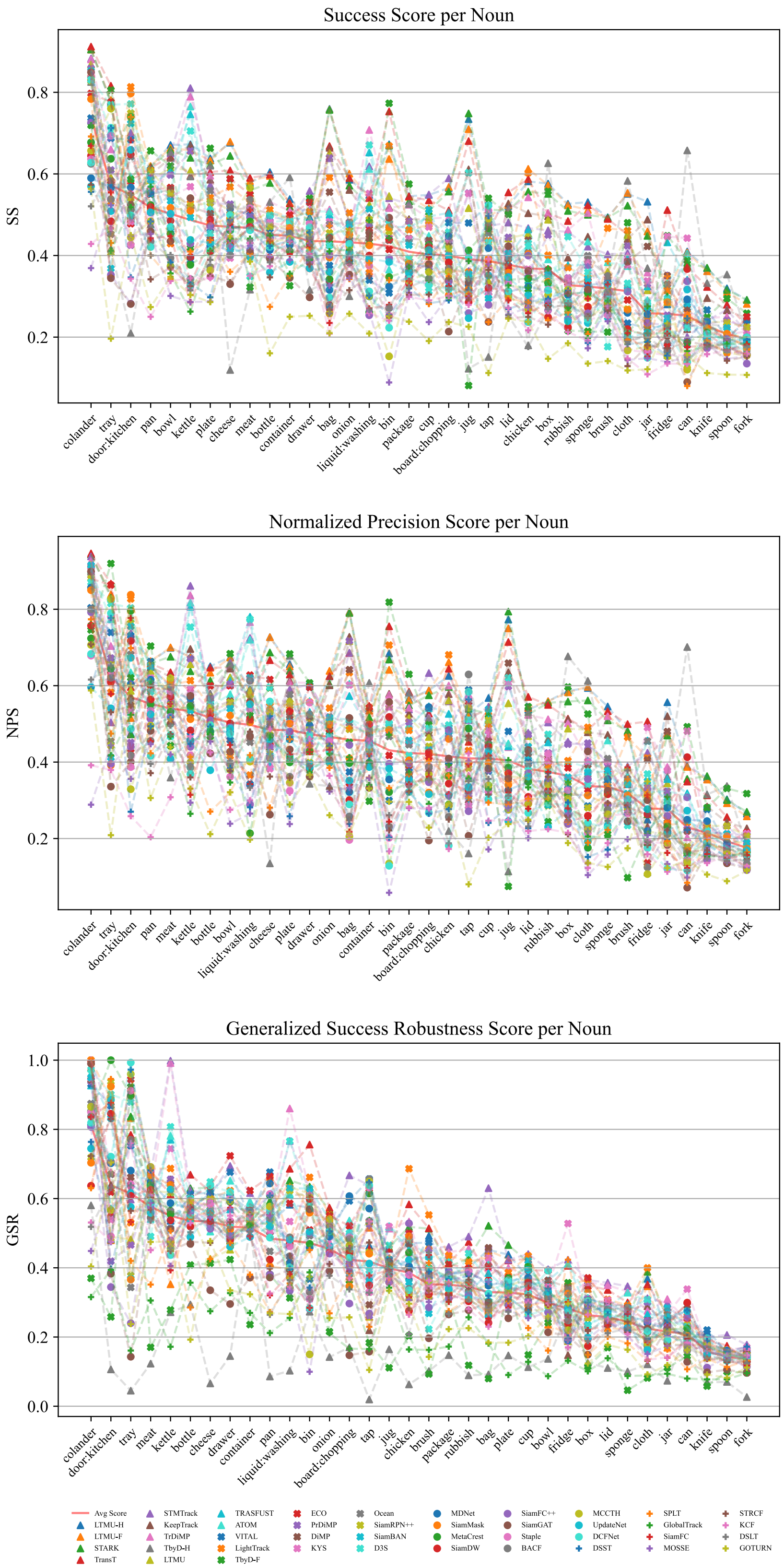

Fig. 23: SS, NPS, and GSR performance achieved under the MSE protocol of the 42 selected trackers with respect to the target nouns available in TREK-150. The red plain line highlights the average tracker performance.
